# Systematic literature review of real-world evidence for treatments in HR+/HER2- second-line LABC/mBC after first-line treatment with CDK4/6i

**DOI:** 10.1186/s12885-024-12269-8

**Published:** 2024-05-23

**Authors:** Veronique Lambert, Sarah Kane, Belal Howidi, Bao-Ngoc Nguyen, David Chandiwana, Yan Wu, Michelle Edwards, Imtiaz A. Samjoo

**Affiliations:** 1 Pfizer, 10017 New York, NY USA; 2 EVERSANA, Burlington, ON Canada; 3 Arvinas, 06511 New Haven, CT USA

**Keywords:** Breast cancer, HR+/HER2-, First-line CDK4/6i, Real-world evidence, Systematic literature review

## Abstract

**Background:**

Cyclin-dependent kinase 4 and 6 inhibitors (CDK4/6i) combined with endocrine therapy (ET) are currently recommended by the National Comprehensive Cancer Network (NCCN) guidelines and the European Society for Medical Oncology (ESMO) guidelines as the first-line (1 L) treatment for patients with hormone receptor-positive, human epidermal growth factor receptor 2-negative, locally advanced/metastatic breast cancer (HR+/HER2- LABC/mBC). Although there are many treatment options, there is no clear standard of care for patients following 1 L CDK4/6i. Understanding the real-world effectiveness of subsequent therapies may help to identify an unmet need in this patient population. This systematic literature review qualitatively synthesized effectiveness and safety outcomes for treatments received in the real-world setting after 1 L CDK4/6i therapy in patients with HR+/ HER2- LABC/mBC.

**Methods:**

MEDLINE®, Embase, and Cochrane were searched using the Ovid® platform for real-world evidence studies published between 2015 and 2022. Grey literature was searched to identify relevant conference abstracts published from 2019 to 2022. The review was conducted in accordance with PRISMA guidelines (PROSPERO registration: CRD42023383914). Data were qualitatively synthesized and weighted average median real-world progression-free survival (rwPFS) was calculated for NCCN/ESMO-recommended post-1 L CDK4/6i treatment regimens.

**Results:**

Twenty records (9 full-text articles and 11 conference abstracts) encompassing 18 unique studies met the eligibility criteria and reported outcomes for second-line (2 L) treatments after 1 L CDK4/6i; no studies reported disaggregated outcomes in the third-line setting or beyond. Sixteen studies included NCCN/ESMO guideline-recommended treatments with the majority evaluating endocrine-based therapy; five studies on single-agent ET, six studies on mammalian target of rapamycin inhibitors (mTORi) ± ET, and three studies with a mix of ET and/or mTORi. Chemotherapy outcomes were reported in 11 studies. The most assessed outcome was median rwPFS; the weighted average median rwPFS was calculated as 3.9 months (3.3-6.0 months) for single-agent ET, 3.6 months (2.5–4.9 months) for mTORi ± ET, 3.7 months for a mix of ET and/or mTORi (3.0–4.0 months), and 6.1 months (3.7–9.7 months) for chemotherapy. Very few studies reported other effectiveness outcomes and only two studies reported safety outcomes. Most studies had heterogeneity in patient- and disease-related characteristics.

**Conclusions:**

The real-world effectiveness of current 2 L treatments post-1 L CDK4/6i are suboptimal, highlighting an unmet need for this patient population.

**Supplementary Information:**

The online version contains supplementary material available at 10.1186/s12885-024-12269-8.

## Introduction

Breast cancer (BC) is the most diagnosed form of cancer in women with an estimated 2.3 million new cases diagnosed worldwide each year [1]. BC is the second leading cause of cancer death, accounting for 685,000 deaths worldwide per year [2]. By 2040, the global burden associated with BC is expected to surpass three million new cases and one million deaths annually (due to population growth and aging) [3]. Numerous factors contribute to global disparities in BC-related mortality rates, including delayed diagnosis, resulting in a high number of BC cases that have progressed to locally advanced BC (LABC) or metastatic BC (mBC) [4–6]. In the United States (US), the five-year survival rate for patients who progress to mBC is three times lower (31%) than the overall five-year survival rate for all stages (91%) [6, 7].

Hormone receptor (HR) positive (i.e., estrogen receptor and/or progesterone receptor positive) coupled with negative human epidermal growth factor 2 (HER2) expression is the most common subtype of BC, accounting for ∼60–70% of all BC cases [8, 9]. Historically, endocrine therapy (ET) through estrogen receptor modulation and/or estrogen deprivation has been the standard of care for first-line (1 L) treatment of HR-positive/HER2-negative (HR+/HER2-) mBC [10]. However, with the approval of the cyclin-dependent kinase 4/6 inhibitor (CDK4/6i) palbociclib in combination with the aromatase inhibitor (AI) letrozole in 2015 by the US Food and Drug Administration (FDA), 1 L treatment practice patterns have evolved such that CDK4/6i (either in combination with AIs or with fulvestrant) are currently considered the standard of care [11–17]. Other CDK4/6i (ribociclib and abemaciclib) in combination with ET are approved for the treatment of HR+/HER2- LABC/mBC; 1 L use of ribociclib in combination with an AI was granted FDA approval in March 2017 for postmenopausal women (with expanded approval in July 2018 for pre/perimenopausal women and for use in 1 L with fulvestrant for patients with disease progression on ET as well as for postmenopausal women), and abemaciclib in combination with fulvestrant was granted FDA approval in September 2017 for patients with disease progression following ET and as monotherapy in cases where disease progression occurs following ET and prior chemotherapy in mBC (with expanded approval in February 2018 for use in 1 L in combination with an AI for postmenopausal women) [18–21].

Clinical trials investigating the addition of CDK4/6i to ET have demonstrated significant improvement in progression-free survival (PFS) and significant (ribociclib) or numerical (palbociclib and abemaciclib) improvement in overall survival (OS) compared to ET alone in patients with HR+/HER2- advanced or mBC, making this combination treatment the recommended option in the 1 L setting [22–27]. However, disease progression occurs in a significant portion of patients after 1 L CDK4/6i treatment [28] and the optimal treatment sequence after progression on CDK4/6i remains unclear [29]. At the time of this review (literature search conducted December 14, 2022), guidelines by the National Comprehensive Cancer Network (NCCN) and the European Society for Medical Oncology (ESMO) recommend various options for the treatment of HR+/HER2- advanced BC in the second-line (2 L) setting, including fulvestrant monotherapy, mammalian target of rapamycin inhibitors (mTORi; e.g., everolimus) ± ET, alpelisib + fulvestrant (if phosphatidylinositol-4,5-bisphosphate 3-kinase catalytic subunit alpha mutation positive [PIK3CA-m+]), poly-ADP ribose polymerase inhibitors (PARPi) including olaparib or talazoparib (if breast cancer gene/partner and localizer of BRCA2 positive [BRCA/PALB2m+]), and chemotherapy (in cases when a visceral crisis is present) [15, 16]. CDK4/6i can also be used in 2 L [16, 30]; however, limited data are available to support CDK4/6i rechallenge after its use in the 1 L setting [15]. Depending on treatments used in the 1 L and 2 L settings, treatment in the third-line setting is individualized based on the patient’s response to prior treatments, tumor load, duration of response, and patient preference [9, 15]. Understanding subsequent treatments after 1 L CDK4/6i, and their associated effectiveness, is an important focus in BC research.

Treatment options for HR+/HER2- LABC/mBC continue to evolve, with ongoing research in both clinical trials and in the real-world setting. Real-world evidence (RWE) offers important insights into novel therapeutic regimens and the effectiveness of treatments for HR+/HER2- LABC/mBC. The effectiveness of the current treatment options following 1 L CDK4/6i therapy in the real-world setting highlights the unmet need in this patient population and may help to drive further research and drug development. In this study, we conducted a systematic literature review (SLR) to qualitatively summarize the effectiveness and safety of treatment regimens in the real-world setting after 1 L treatment with CDK4/6i in patients with HR+/HER2- LABC/mBC.

## Methods

### Literature search

An SLR was performed in accordance with the Cochrane Handbook for Systematic Reviews of Interventions [31] and reported in alignment with the Preferred Reporting Items for Systematic Literature Reviews and Meta-Analyses (PRISMA) statement [32] to identify all RWE studies assessing the effectiveness and safety of treatments used for patients with HR+/HER2- LABC/mBC following 1 L CDK4/6i therapy and received subsequent treatment in 2 L and beyond (2 L+). The Ovid® platform was used to search MEDLINE® (including Epub Ahead of Print and In-Process, In-Data-Review & Other Non-Indexed Citations), Ovid MEDLINE® Daily, Embase, Cochrane Central Register of Controlled Trials, and Cochrane Database of Systematic Reviews by an experienced medical information specialist. The MEDLINE® search strategy was peer-reviewed independently by a senior medical information specialist before execution using the Peer Review of Electronic Search Strategies (PRESS) checklist [33]. Searches were conducted on December 14, 2022. The review protocol was developed *a priori* and registered with the International Prospective Register of Systematic Review (PROSPERO; CRD42023383914) which outlined the population, intervention, comparator, outcome, and study design (PICOS) criteria and methodology used to conduct the review (Table 1).

Search strategies utilized a combination of controlled vocabulary (e.g., “HER2 Breast Cancer” or “HR Breast Cancer”) and keywords (e.g., “Retrospective studies”). Vocabulary and syntax were adjusted across databases. Published and validated filters were used to select for study design and were supplemented using additional medical subject headings (MeSH) terms and keywords to select for RWE and nonrandomized studies [34]. No language restrictions were included in the search strategy. Animal-only and opinion pieces were removed from the results. The search was limited to studies published between January 2015 and December 2022 to reflect the time at which FDA approval was granted for the first CDK4/6i agent (palbociclib) in combination with AI for the treatment of LABC/mBC [35]. Further search details are presented in **Supplementary Material 1**.

Grey literature sources were also searched to identify relevant abstracts and posters published from January 2019 to December 2022 for prespecified relevant conferences including ESMO, San Antonio Breast Cancer Symposium (SABCS), American Society of Clinical Oncology (ASCO), the International Society for Pharmacoeconomics and Outcomes Research (ISPOR US), and the American Association for Cancer Research (AACR). A search of ClinicalTrials.gov was conducted to validate the findings from the database and grey literature searches.

### Study selection, data extraction & weighted average calculation

Studies were screened for inclusion using DistillerSR Version 2.35 and 2.41 (DistillerSR Inc. 2021, Ottawa, Canada) by two independent reviewers based on the prespecified PICOS criteria (Table 1). A third reviewer was consulted to resolve any discrepancies during the screening process. Studies were included if they reported RWE on patients aged ≥ 18 years with HR+/HER2- LABC/mBC who received 1 L CDK4/6i treatment and received subsequent treatment in 2 L+. Studies were excluded if they reported the results of clinical trials (i.e., non-RWE), were published in any language other than English, and/or were published prior to 2015 (or prior to 2019 for conference abstracts and posters). For studies that met the eligibility criteria, data relating to study design and methodology, details of interventions, patient eligibility criteria and baseline characteristics, and outcome measures such as efficacy, safety, tolerability, and patient-reported outcomes (PROs), were extracted (as available) using a Microsoft Excel®-based data extraction form (Microsoft Corporation, WA, USA). Data extraction was performed by a single reviewer and was confirmed by a second reviewer. Multiple publications identified for the same RWE study, patient population, and setting that reported data for the same intervention were linked and extracted as a single publication. Weighted average median real-world progression-free survival (rwPFS) values were calculated by considering the contribution to the median rwPFS of each study proportional to its respective sample size. These weighted values were then used to compute the overall median rwPFS estimate.

### Quality assessment

The Newcastle-Ottawa scale (NOS) for nonrandomized (cohort) studies was used to assess the risk of bias for published, full-text studies [36]. The NOS allocates a maximum of nine points for the least risk of bias across three domains: (1) Formation of study groups (four points), (2) Comparability between study groups (two points), (3) Outcome ascertainment (three points). NOS scores can be categorized in three groups: very high risk of bias (0 to 3 points), high risk of bias (4 to 6), and low risk of bias (7 to 9) [37]. Risk of bias assessment was performed by one reviewer and validated by a second independent reviewer to verify accuracy. Due to limited methodological data by which to assess study quality, risk of bias assessment was not performed on conference abstracts or posters. An amendment to the PROSPERO record (CRD42023383914) for this study was submitted in relation to the quality assessment method (specifying usage of the NOS).

**Table 1:** PICOS criteria

	Inclusion Criteria	Exclusion Criteria
*Population*	Patients aged ≥ 18 years with HR+/HER2- LABC/mBC who received CDK4/6i treatment in 1 L and received treatment for BC in 2 L+	• Non-human • Age < 18 years • Unconfirmed diagnosis of HR+/HER2- LABC/mBC • 1 L therapy only • Did not receive CDK4/6i in 1 L (including patient groups with mixed lines of CDK4/6i treatment)
*Interventions/* *Comparators*	• Pharmacologic interventions • Any not excluded	• Treatments not related to HR+/HER2- LABC/mBC • Non-pharmacological interventions (e.g., medical devices, lifestyle alteration regimens) • Alternative medicines
*Outcomes*	Effectiveness outcomes: • Clinical benefit rate [CBR], objective response rate [ORR], complete/partial response [CR/PR], duration of response [DOR], time-to-next-treatment [TTNT], time-to-progression [TTP], progression-free survival [PFS], overall survival [OS] Safety outcomes: • Overall rate of adverse events [AEs], AEs of grade 3–4 severity, discontinuation due to AEs • Treatment emergent adverse effects Tolerability outcomes: • Treatment duration, modifications, discontinuations Patient-reported outcomes: • Quality of life/utility (e.g., physical symptoms, psychological distress)	• Any not listed as inclusion
*Study Design* ^*a*^	• RWE studies (observational, prospective or retrospective studies) • Published articles (January 2015 to December 2022) • Conference abstracts/posters (January 2019 to December 2022)	• Any non-RWE studies • Articles published prior to 2015 • Conference abstracts/posters published before 2019
*Restrictions*	• Articles in English^b^	• All non-English articles

^a^ Relevant conference abstracts include SABCS, ASCO, ESMO, ISPOR US, and AACR.

^b^ Citation retrieval was not limited by language. Non-English records were excluded during the abstract and full-text screening stages. Abbreviations: 1 L = first-line; 2 L + = second-line and beyond; AACR = American Association of Cancer Research; AE = adverse event; ASCO = American Society of Clinical Oncology; BC = breast cancer; CBR = clinical benefit rate; CDK4/6i = cyclin-dependent kinase 4/6 inhibitor; CR = complete response; DOR = duration of response; ESMO = European Society for Medical Oncology; HER2 = human epidermal growth factor receptor 2; HR = hormone receptor; ISPOR US = Professional Society for Health Economics and Outcomes Research in the United States; LABC = locally advanced breast cancer; mBC = metastatic breast cancer; ORR = objective response rate; OS = overall survival; PFS = progression-free survival; PR = partial response; RWE = real-world evidence; SABCS = San Antonio Breast Cancer Symposium; SLR = systematic literature review; TTNT = time-to-next-treatment; TTP = time-to-progression.

## Results

### Literature search

**Fig. 1:**
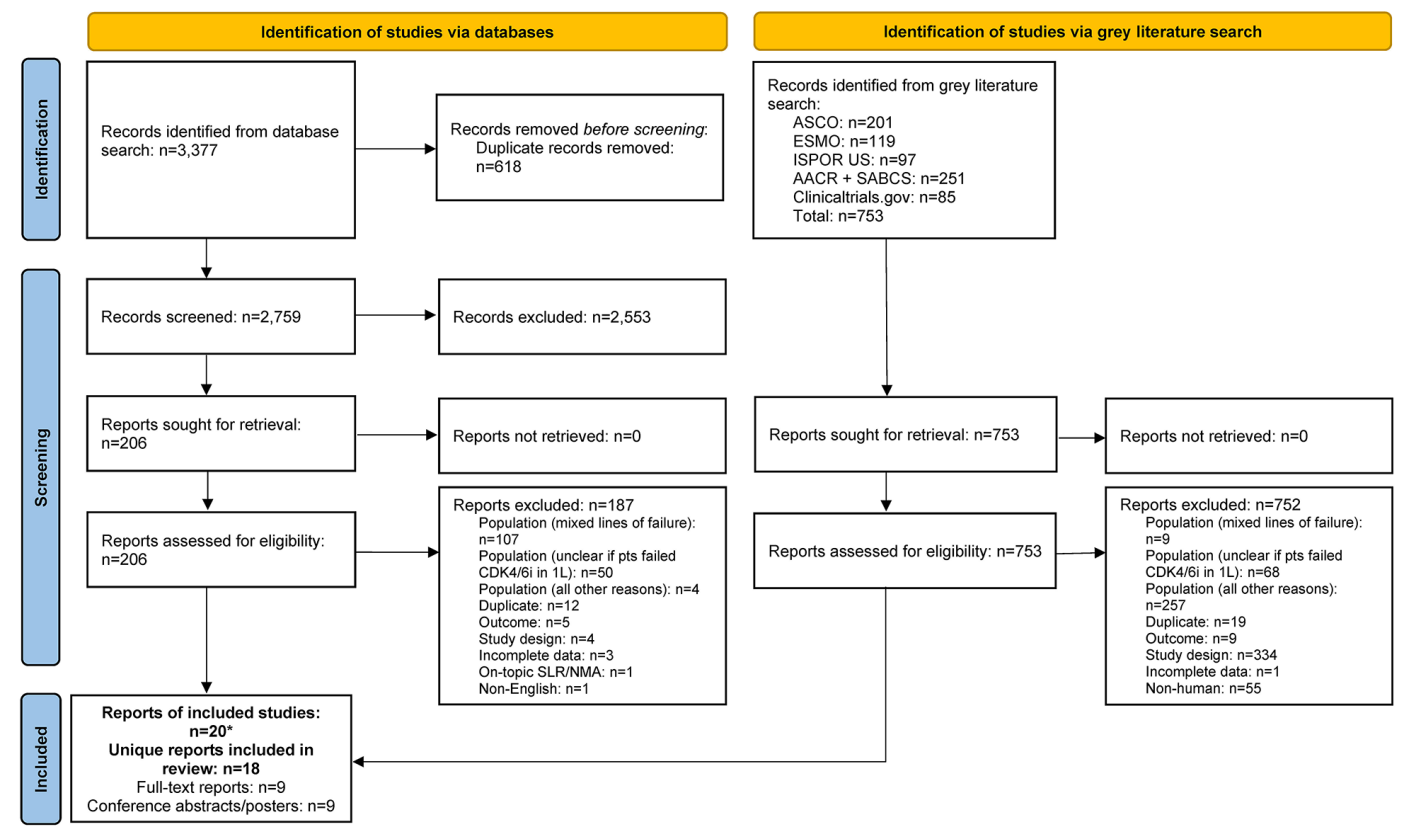
PRISMA flow diagram. *Two included conference abstracts reported the same information as already included full-text reports, hence both conference abstracts were not identified as unique. Abbreviations: 1 L = first-line; AACR = American Association of Cancer Research; ASCO = American Society of Clinical Oncology; CDK4/6i = cyclin-dependent kinase 4/6 inhibitor; ESMO = European Society for Medical Oncology; ISPOR = Professional Society for Health Economics and Outcomes Research; n = number of studies; NMA = network meta-analysis; pts = participants; SABCS = San Antonio Breast Cancer Symposium; SLR = systematic literature review.

The database search identified 3,377 records; after removal of duplicates, 2,759 were screened at the title and abstract stage of which 2,553 were excluded. Out of the 206 reports retrieved and assessed for eligibility, an additional 187 records were excluded after full-text review; most of these studies were excluded for having patients with mixed lines of CDK4/6i treatment (i.e., did not receive CDK4/6i exclusively in 1 L) (Fig. 1 and Table S1). The grey literature search identified 753 records which were assessed for eligibility; of which 752 were excluded mainly due to the population not meeting the eligibility criteria (Fig. 1). In total, the literature searches identified 20 records (9 published full-text articles and 11 conference abstracts/posters) representing 18 unique RWE studies that met the inclusion criteria. The NOS quality scores for the included full-text articles are provided in Table S2. The scores ranged from four to six points (out of a total score of nine) and the median score was five, indicating that all the studies suffered from a high risk of bias [37].

Most studies were retrospective analyses of chart reviews or medical registries, and all studies were published between 2017 and 2022 (Table S3). Nearly half of the RWE studies (8 out of 18 studies) were conducted in the US [38–45], while the remaining studies included sites in Canada, China, Germany, Italy, Japan, and the United Kingdom [46–54]. Sample sizes ranged from as few as 4 to as many as 839 patients across included studies, with patient age ranging from 26 to 86 years old.

Although treatment characteristics in the 1 L setting were not the focus of the present review, these details are captured in Table S3. Briefly, several RWE studies reported 1 L CDK4/6i use in combination with ET (8 out of 18 studies) or as monotherapy (2 out of 18 studies) (Table S3). Treatments used in combination with 1 L CDK4/6i included letrozole, fulvestrant, exemestane, and anastrozole. Where reported (4 out of 18 studies), palbociclib was the most common 1 L CDK4/6i treatment. Many studies (8 out of 18 studies) did not report which specific CDK4/6i treatment(s) were used in 1 L or if its administration was in combination or monotherapy.

### Characteristics of treatments after 1 L CDK4/6i therapy

Across all studies included in this review, effectiveness and safety data were only available for treatments administered in the 2 L setting after 1 L CDK4/6i treatment. No studies were identified that reported outcomes for patients treated in the third-line setting or beyond after 1 L CDK4/6i treatment. All 18 studies reported effectiveness outcomes in 2 L, with only two of these studies also describing 2 L safety outcomes. The distribution of outcomes reported in these studies is provided in Table S4. Studies varied in their reporting of outcomes for 2 L treatments; some studies reported outcomes for a group of 2 L treatments while others described independent outcomes for specific 2 L treatments (i.e., everolimus, fulvestrant, or chemotherapy agents such as eribulin mesylate) [42, 45, 50, 54, 55]. Due to the heterogeneity in treatment classes reported in these studies, this data was categorized (as described below) to align with the guidelines provided by NCCN and ESMO [15, 16]. The treatment class categorizations for the purpose of this review are: **single-agent ET** (patients who exclusively received a single-agent ET after 1 L CDK4/6i treatment), **mTORi ± ET** (patients who exclusively received an mTORi with or without ET after 1 L CDK4/6i treatment), **mix of ET and/or mTORi** (patients who may have received only ET, only mTORi, and/or both treatments but the studies in this group lacked sufficient information to categorize these patients in the “single-agent ET” or “mTOR ± ET” categories), and **chemotherapy** (patients who exclusively received chemotherapy after 1 L CDK4/6i treatment). Despite ESMO and NCCN guidelines indicating that limited evidence exists to support rechallenge with CDK4/6i after 1 L CDK4/6i treatment [15, 16], two studies reported outcomes for this treatment approach. Data for such patients were categorized as “**CDK4/6i ± ET**” as it was unclear how many patients receiving CDK4/6i rechallenge received concurrent ET. All other patient groups that lacked sufficient information or did not report outcome/safety data independently (i.e., grouped patients with mixed treatments) to categorize as one of the treatment classes described above were grouped as “**other**”.

**Fig. 2:**
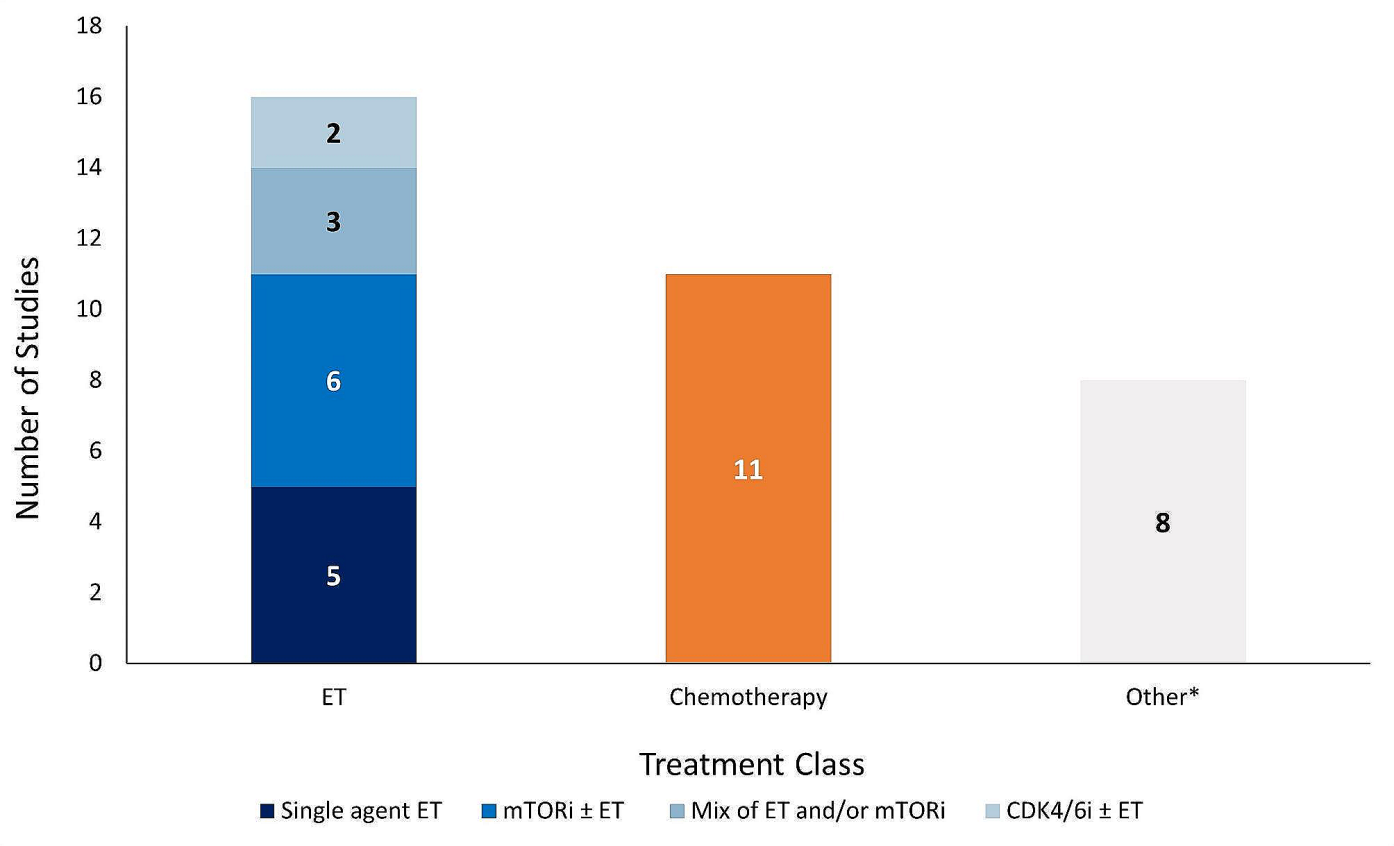
Number of studies reporting effectiveness outcomes exclusively for each treatment class. *Studies that lack sufficient information on effectiveness outcomes to classify based on the treatment classes outlined in the legend above. Abbreviations: CDK4/6i = cyclin-dependent kinase 4/6 inhibitor; ET = endocrine therapy; mTORi = mammalian target of rapamycin inhibitor.

The majority of studies reported effectiveness outcomes for endocrine-based therapy after 1 L CDK4/6i treatment; five studies for single-agent ET, six studies for mTORi ± ET, and three studies for a mix of ET and/or mTORi (Fig. 2). Eleven studies reported effectiveness outcomes for chemotherapy after 1 L CDK4/6i treatment, and only two studies reported effectiveness outcomes for CDK4/6i rechallenge ± ET. Eight studies that described effectiveness outcomes were grouped into the “other” category. Safety data was only reported in two studies: one study evaluating the chemotherapy agent eribulin mesylate and one evaluating the mTORi everolimus.

### Effectiveness outcomes

#### Real-world progression-free survival

Median rwPFS was described in 13 studies (Tables 2 and Table S5). Across the 13 studies, the median rwPFS ranged from 2.5 months [49] to 17.3 months [39]. Out of the 13 studies reporting median rwPFS, 10 studies reported median rwPFS for a 2 L treatment recommended by ESMO and NCCN guidelines, which ranged from 2.5 months [49] to 9.7 months [45].

**Fig. 3:**
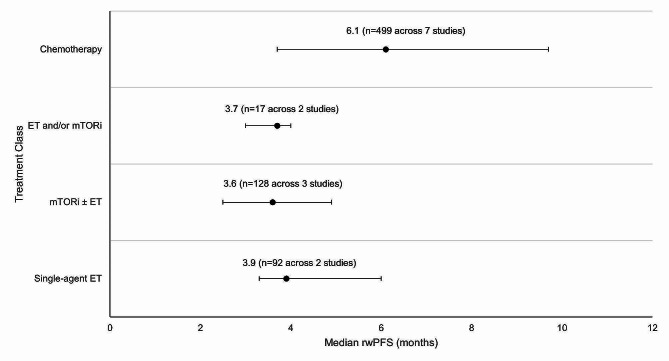
Weighted average median rwPFS for 2 L treatments (recommended in ESMO/NCCN guidelines) after 1 L CDK4/6i treatment. Circular dot represents weighted average median across studies. Horizontal bars represent the range of values reported in these studies. Abbreviations: CDK4/6i = cyclin-dependent kinase 4/6 inhibitor; ESMO = European Society for Medical Oncology; ET = endocrine therapy, mTORi = mammalian target of rapamycin inhibitor; n = number of patients; NCCN = National Comprehensive Cancer Network; rwPFS = real-world progression-free survival.

Weighted average median rwPFS was calculated for 2 L treatments recommended by both ESMO and NCCN guidelines (Fig. 3). The weighted average median rwPFS for single-agent ET was 3.9 months (*n* = 92 total patients) and was derived using data from two studies reporting median rwPFS values of 3.3 months (*n* = 70) [38] and 6.0 months (*n* = 22) [40]. For one study (*n* = 7) that reported outcomes for single agent ET, median rwPFS was not reached during the follow-up period; as such, this study was excluded from the weighted average median rwPFS calculation [49].

The weighted average median rwPFS for mTORi ± ET was 3.6 months (*n* = 128 total patients) and was derived based on data from 3 studies with median rwPFS ranging from 2.5 months (*n* = 4) [49] to 4.9 months (*n* = 25) [54] (Fig. 3). For patients who received a mix of ET and/or mTORi but could not be classified into the single-agent ET or mTORi ± ET treatment classes, the weighted average median rwPFS was calculated to be 3.7 months (*n* = 17 total patients). This was calculated based on data from two studies reporting median rwPFS values of 3.0 months (*n* = 5) [46] and 4.0 months (*n* = 12) [49]. Notably, one study of patients receiving ET and/or everolimus reported a median rwPFS duration of 3.0 months; however, this study was excluded from the weighted average median rwPFS calculation for the ET and/or mTORi class as the sample size was not reported [53].

The weighted average median rwPFS for chemotherapy was 6.1 months (*n* = 499 total patients), calculated using data from 7 studies reporting median rwPFS values ranging from 3.7 months (*n* = 249) [38] to 9.7 months (*n* = 121) [45] (Fig. 3). One study with a median rwPFS duration of 5.6 months was not included in the weighted average median rwPFS calculation as the study did not report the sample size [53]. A second study was excluded from the calculation since the reported median rwPFS was not reached during the study period (*n* = 7) [41].

Although 2 L CDK4/6i ± ET rechallenge lacks sufficient information to support recommendation by ESMO and NCCN guidelines, the limited data currently available for this treatment have shown promising results. Briefly, two studies reported median rwPFS for CDK4/6i ± ET with values of 8.3 months (*n* = 302) [38] and 17.3 months (*n* = 165) (Table 2) [39]. The remaining median rwPFS studies reported data for patients classified as “Other” (Table S5). The “Other” category included median rwPFS outcomes from seven studies, and included a myriad of treatments (e.g., ET, mTOR + ET, chemotherapy, CDK4/6i + ET, alpelisib + fulvestrant, chidamide + ET) for which disaggregated median rwPFS values were not reported.

#### Overall survival

Median OS for 2 L treatment was reported in only three studies (Table 2) [38, 42, 43]. Across the three studies, the 2 L median OS ranged from 5.2 months (*n* = 3) [43] to 35.7 months (*n* = 302) [38]. Due to the lack of OS data in most of the studies, weighted averages could not be calculated. No median OS data was reported for the single-agent ET treatment class whereas two studies reported median OS for the mTORi ± ET treatment class, ranging from 5.2 months (*n* = 3) [43] to 21.8 months (*n* = 54) [42]. One study reported 2 L median OS of 24.8 months for a single patient treated with chemotherapy [43]. The median OS data in the CDK4/6i ± ET rechallenge group was 35.7 months (*n* = 302) [38].

Patient mortality was reported in three studies [43–45]. No studies reported mortality for the single-agent ET treatment class and only one study reported this outcome for the mTORi ± ET treatment class, where 100% of patients died (*n* = 3) as a result of rapid disease progression [43]. For the chemotherapy class, one study reported mortality for one patient receiving 2 L capecitabine [43]. An additional study reported eight deaths (21.7%) following 1 L CDK4/6i treatment; however, this study did not disclose the 2 L treatments administered to these patients [44].

#### Other clinical endpoints

The studies included limited information on additional clinical endpoints; two studies reported on time-to-discontinuation (TTD), two reported on duration of response (DOR), and one each on time-to-next-treatment (TTNT), time-to-progression (TTP), objective response rate (ORR), clinical benefit rate (CBR), and stable disease (Tables 2 and Table S5).

### Safety, tolerability, and patient-reported outcomes

Safety and tolerability data were reported in two studies [40, 45]. One study investigating 2 L administration of the chemotherapy agent eribulin mesylate reported 27 patients (22.3%) with neutropenia, 3 patients (2.5%) with febrile neutropenia, 10 patients (8.3%) with peripheral neuropathy, and 14 patients (11.6%) with diarrhea [45]. Of these, neutropenia of grade 3–4 severity occurred in 9 patients (33.3%) [45]. A total of 55 patients (45.5%) discontinued eribulin mesylate treatment; 1 patient (0.83%) discontinued treatment due to adverse events [45]. Another study reported that 5 out of the 22 patients receiving the mTORi everolimus combined with ET in 2 L (22.7%) discontinued treatment due to toxicity [40]. PROs were not reported in any of the studies included in the SLR.

## Discussion

The objective of this study was to summarize the existing RWE on the effectiveness and safety of therapies for patients with HR+/HER2- LABC/mBC after 1 L CDK4/6i treatment. We identified 18 unique studies reporting specifically on 2 L treatment regimens after 1 L CDK4/6i treatment. The weighted average median rwPFS for NCCN- and ESMO- guideline recommended 2 L treatments ranged from 3.6 to 3.9 months for ET-based treatments and was 6.1 months when including chemotherapy-based regimens. Treatment selection following 1 L CDK4/6i therapy remains challenging primarily due to the suboptimal effectiveness or significant toxicities (e.g., chemotherapy) associated with currently available options [56]. These results highlight that currently available 2 L treatments for patients with HR+/HER2- LABC/mBC who have received 1 L CDK4/6i are suboptimal, as evidenced by the brief median rwPFS duration associated with ET-based treatments, or notable side effects and toxicity linked to chemotherapy. This conclusion is aligned with a recent review highlighting the limited effectiveness of treatment options for HR+/HER2- LABC/mBC patients post-CDK4/6i treatment [56, 57]. Registrational trials which have also shed light on the short median PFS of 2–3 months achieved by ET (i.e., fulvestrant) after 1 L CDK4/6i therapy emphasize the need to develop improved treatment strategies aimed at prolonging the duration of effective ET-based treatment [56].

The results of this review reveal a paucity of additional real-world effectiveness and safety evidence after 1 L CDK4/6i treatment in HR+/HER2- LABC/mBC. OS and DOR were only reported in two studies while other clinical endpoints (i.e., TTD, TTNT, TTP, ORR, CBR, and stable disease) were only reported in one study each. Similarly, safety and tolerability data were only reported in two studies each, and PROs were not reported in any study. This hindered our ability to provide a comprehensive assessment of real-world treatment effectiveness and safety following 1 L CDK4/6i treatment. The limited evidence may be due to the relatively short period of time that has elapsed since CDK4/6i first received US FDA approval for 1 L treatment of HR+/HER2- LABC/mBC (2015) [35]. As such, almost half of our evidence was informed by conference abstracts. Similarly, no real-world studies were identified in our review that reported outcomes for treatments in the third- or later-lines of therapy after 1 L CDK4/6i treatment. The lack of data in this patient population highlights a significant gap which limits our understanding of the effectiveness and safety for patients receiving later lines of therapy. As more patients receive CDK4/6i therapy in the 1 L setting, the number of patients requiring subsequent lines of therapy will continue to grow. Addressing this data gap over time will be critical to improve outcomes for patients with HR+/HER2- LABC/mBC following 1 L CDK4/6i therapy.

There are several strengths of this study, including adherence to the guidelines outlined in the Cochrane Handbook to ensure a standardized and reliable approach to the SLR [58] and reporting of the SLR following PRISMA guidelines to ensure transparency and reproducibility [59]. Furthermore, the inclusion of only RWE studies allowed us to assess the effectiveness of current standard of care treatments outside of a controlled environment and enabled us to identify an unmet need in this patient population.

This study had some notable limitations, including the lack of safety and additional effectiveness outcomes reported. In addition, the dearth of studies reporting PROs is a limitation, as PROs provide valuable insight into the patient experience and are an important aspect of assessing the impact of 2 L treatments on patients’ quality of life. The studies included in this review also lacked consistent reporting of clinical characteristics (e.g., menopausal status, sites of metastasis, prior surgery) making it challenging to draw comprehensive conclusions or comparisons based on these factors across the studies. Taken together, there exists an important gap in our understanding of the long-term management of patients with HR+/HER2- LABC/mBC. Additionally, the effectiveness results reported in our evidence base were informed by small sample sizes; many of the included studies reported median rwPFS based on less than 30 patients [39–41, 46, 49, 51, 60], with two studies not reporting the sample size at all [47, 53]. This may impact the generalizability and robustness of the results. Relatedly, the SLR database search was conducted in December 2022; as such, novel agents (e.g., elacestrant and capivasertib + fulvestrant) that have since received FDA approval for the treatment of HR+/HER2- LABC/mBC may impact current 2 L rwPFS outcomes [61, 62]. Finally, relative to the number of peer-reviewed full-text articles, this SLR identified eight abstracts and one poster presentation, comprising half (50%) of the included unique studies. As conference abstracts are inherently limited by how much content that can be described due to word limit constraints, this likely had implications on the present synthesis whereby we identified a dearth of real-world effectiveness outcomes in patients with HR+/HER2- LABC/mBC treated with 1 L CDK4/6i therapy.

Future research in this area should aim to address the limitations of the current literature and provide a more comprehensive understanding of optimal sequencing of effective and safe treatment for patients following 1 L CDK4/6i therapy. Specifically, future studies should strive to report robust data related to effectiveness, safety, and PROs for patients receiving 2 L treatment after 1 L CDK4/6i therapy. Future studies should also aim to understand the mechanism underlying CDK4/6i resistance. Addressing these gaps in knowledge may improve the long-term real-world management of patients with HR+/HER2- LABC/mBC. A future update of this synthesis may serve to capture a wider breadth of full-text, peer-reviewed articles to gain a more robust understanding of the safety, effectiveness, and real-world treatment patterns for patients with HR+/HER2- LABC/mBC. This SLR underscores the necessity for ongoing investigation and the development of innovative therapeutic approaches to address these gaps and improve patient outcomes.

## Conclusion

This SLR qualitatively summarized the existing real-world effectiveness data for patients with HR+/HER2- LABC/mBC after 1 L CDK4/6i treatment. Results of this study highlight the limited available data and the suboptimal effectiveness of treatments employed in the 2 L setting and underscore the unmet need in this patient population. Additional studies reporting effectiveness and safety outcomes, in addition to PROs, for this patient population are necessary and should be the focus of future research.

**Table 2:** Second-line effectiveness outcomes for patients who received first-line CDK4/6i treatment in real-world evidence studies

Second-line treatment	Median rwPFS, months (n patients)	Other clinical endpoints^a^, months (unless otherwise specified) (n patients)	Median OS, months (n patients)
Single-agent ET^b^	**3.3-6.0 across 2 studies**: 3.3 in 1 article (38) (*n* = 70) 6.0 in 1 article (40) (*n* = 22) Not reached in 1 abstract (49) (*n* = 7)	**TTD: 3.1–3.4 across 2 studies**: 3.1 in 1 abstract (44) (*n* = 11) 3.4 in 1 abstract (55) (*n* = 88)	NR
mTORi ± ET	**2.5–4.9 across 3 studies**: 2.5^c^ in 1 abstract (49) (*n* = 4) 3.3 in 1 article (38) (*n* = 99) 4.9 in 1 abstract (54) (*n* = 25)	**TTNT**: 4.3 in 1 article (42) (*n* = 54) **TTD**: 13.2 in 1 abstract (44) (*n* = 10) **TTP**: 2.1 in 1 article (43) (*n* = 3)	**5.2–21.8 across 2 studies**: 5.2 in 1 article (43) (*n* = 3) 21.8 in 1 article (42) (*n* = 54)
Mix of ET and/or mTORi	**3.0–4.0 across 3 studies**: 3.0 in 1 abstract (53) (n = NR^d^; ET and/or everolimus, not further defined) 3.0 in 1 abstract (46) (*n* = 5; everolimus or fulvestrant) 4.0^c^ in 1 abstract (49) (*n* = 12; 7 fulvestrant, 4 everolimus + exemestane, 1 tamoxifen)	NR	NR
Chemotherapy	**3.7–9.7 across 7 studies**: 3.7 in 1 article (38) (*n* = 249) 5.4 in 1 article (40) (*n* = 11) 5.4^c^ in 1 abstract (49) (*n* = 22) 5.6 in 1 abstract (53) (n = NR^d^) 6.0 in 1 abstract (46) (*n* = 20) 7.2 in 1 article (51) (*n* = 22) 9.0^e^ in 1 article (52) (*n* = 54) 9.7 in 1 article (45) (*n* = 121) Not reached in 1 article (41) (*n* = 7)	**TTP**: 2.8 in 1 article (43) (*n* = 1) **TTD**: 4.1 in 1 abstract (44) (*n* = 8) **ORR**: 42.2% in 1 article (45) (*n* = 51) **CBR**: 58.7% in 1 article (45) (*n* = 71) **Stable disease**: 16.5% in 1 article (45) (*n* = 20) **DOR**: 4.7 in 1 article (45) (*n* = 121)	24.8 in 1 article (43) (*n* = 1)
CDK4/6i ± ET (rechallenge)	**8.3–17.3 across 2 studies**: 8.3 in 1 article (38) (*n* = 302) 17.3 in 1 abstract (39) (*n* = 165)	NR	35.7 in 1 article (38) (*n* = 302)

^a^ Includes clinical outcomes not captured in other columns (i.e., median TTP, median TTNT, median TTD, median DOR, ORR, CBR, and stable disease). ^b^ ET includes AIs (anastrozole, letrozole, and exemestane), selective estrogen receptor modulators (tamoxifen, toremifene, endoxifen), or selective estrogen receptor degraders (fulvestrant). ^c^ Study reports values in weeks which have been converted to months [49]. ^d^ Study did not report sample size [53]. ^e^ Although this study reports this information as TTP, it has been extracted as rwPFS since the definition includes death, not only progression [52]. Abbreviations: AI = aromatase inhibitor; CBR = clinical benefit rate; CDK4/6i = cyclin-dependent kinase 4/6 inhibitor; DOR = duration of response; ET = endocrine therapy; mTORi = mammalian target of rapamycin inhibitor; n = number of patients; NR = not reported; ORR = objective response rate; OS = overall survival; rwPFS = real-world progression-free survival; TTD = time-to-discontinuation; TTNT = time-to-next-treatment; TTP = time-to-progression.

### Electronic supplementary material

Below is the link to the electronic supplementary material.

Supplementary Material 1

Supplementary Material 2

## Data Availability

All data generated or analyzed during this study are included in this published article [and its supplementary information files]. This study is registered with PROSPERO (CRD42023383914).

## Abbreviations

1L: First-line
2L: Second-line
2L+: Second-line treatment setting and beyond
AACR: American Association of Cancer Research
AI: Aromatase inhibitor
ASCO: American Society of Clinical Oncology
BC: Breast cancer
BRCA/PALB2m+: breast cancer gene/partner and localizer of BRCA2 positive
CBR: Clinical benefit rate
CDK4/6i: Cyclin-dependent kinase 4/6 inhibitor
CR: Complete response
DOR: Duration of response
ESMO: European Society for Medical Oncology
FDA: Food and Drug Administration
HER2: Human epidermal growth factor receptor 2
HER2-: Human epidermal growth factor receptor 2 negative
HR: Hormone receptor
HR+: Hormone receptor positive
ISPOR US: Professional Society for Health Economics and Outcomes Research
LABC: Locally advanced breast cancer
mBC: Metastatic breast cancer
MEDLINE: Medical Literature Analysis and Retrieval System Online
MeSH: Medical subject headings
mTORi: Mammalian target of rapamycin inhibitor
NCCN: National Comprehensive Cancer Network
NOS: Newcastle Ottawa Scale
ORR: Objective response rate
OS: Overall survival
PARPi: Poly-ADP ribose polymerase inhibitor
PFS: Progression-free survival
PICOS: Population, Intervention, Comparator, Outcome, Study Design
PR: Partial response
PRISMA: Preferred Reporting Items for Systematic Literature Reviews and Meta-Analyses
PROs: Patient-reported outcomes
RWE: Real-world evidence
rwPFS: Real-world progression-free survival
SABCS: San Antonio Breast Cancer Symposium
SLR: Systematic literature review
TTD: Time-to-discontinuation
TTNT: Time-to-next-treatment
TTP: Time-to-progression
US: United States
