# Supplementary material for: Systematic literature review of real-world evidence for treatments in HR+/HER2- second-line LABC/mBC after first-line treatment with CDK4/6i
Source: BMC Cancer. 2024 May 23;24:631. doi: 10.1186/s12885-024-12269-8 (PMC11112888; doi:10.1186/s12885-024-12269-8)
Supplement: Supplementary file 1 — Supplementary Material 1 [file 12885_2024_12269_MOESM1_ESM.docx]

**Information Specialist performing search :** Joanna Bielecki [Joanna.Bielecki@Eversana.com](mailto:Joanna.Bielecki@Eversana.com)

**Date of the search:** 14 Dec 2022

**Databases searched:**

Ovid MEDLINE(R) and Epub Ahead of Print, In-Process & Other Non-Indexed Citations and Daily Ovid EMBASE

Ovid EBM Reviews - Cochrane Central Register of Controlled Trials

Ovid EBM Reviews - Cochrane Database of Systematic Reviews

**Limits:**

Adults

Humans

Opinion publications removed

Last 3 years of conference abstracts retained (in Embase)

Date limit: 2015 – Current

**Filters:**

Fraser C, Murray A, Burr J. [Identifying observational studies of surgical interventions in MEDLINE and EMBASE](http://www.biomedcentral.com/1471-2288/6/41). *BMC Med Res Methodol.* 2006;6(41).

Search history saved in Corner3 account as: 1799 - Breast Cancer mBC HRplus-HER2minus - 2L-plus and CDK4 6i drugs - MEDLINE Embase CENTRAL CDSR - FINAL

**MULTIFILE SEARCH**

Database(s): **EBM Reviews - Cochrane Central Register of Controlled Trials**November 2022**, EBM Reviews - Cochrane Database of Systematic Reviews**2005 to December 7, 2022**, Embase**1974 to 2022 December 13**, Ovid MEDLINE(R) and Epub Ahead of Print, In-Process, In-Data-Review & Other Non-Indexed Citations and Daily**1946 to December 13, 2022
Search Strategy:

| **#** | **Searches** | **Results** |
| --- | --- | --- |
| 1 | exp Breast Neoplasms/ or exp Breast Carcinoma In Situ/ or (((breast$ or mamma or mammary) adj3 (adenocarcinoma$ or cancer$ or carcinoma$ or neoplasm$ or tumour$ or tumor$ or malignan$)) or ((ductal or duct or intraductal or intra-ductal) adj1 (carcinoma? or hyper-plasia? or hyperplasia?)) or (lobul$ carcinoma? adj2 "in situ") or (paget$ and (areola? or breast$ or mammary or nipple$)) or ((IBC or DCIS or LCIS) and (breast$ or mamma or mammary))).tw,kf,kw,ot. [BREAST CANCER] | 1239688 |
| 2 | Receptor, ErbB-2/ or ErbB Receptors/ or (ErbB2 or "ErbB 2" or HER2$ or "HER 2$" or "c-ErbB2" or "C-ErbB 2" or ((oncoprotein$ or onco-protein$ or protein$ or receptor$) adj1 (neu or neuregulin)) or CD340 or "p185(c-neu)" or p185erbB or "neu protooncogene" or "neu proto-oncogene" or NGL or "metastatic lymph node gene 19" or MLN19 or MLN 19 or "epidermal growth factor receptor 2" or EGFR2 or "EGFR 2" or "HR+/HER2+" or "HR+/HER 2+" or "ER+/HER2+" or "ER+/HER 2+" or "HR+/HER2-" or "HR+/HER 2-" or "ER+/HER2-" or "ER+/HER 2-").tw,kf,kw,ot. [Erb-b2 RECEPTOR TERMS] | 307884 |
| 3 | 1 and 2 [HER2 BREAST CANCER] | 137745 |
| 4 | exp Receptors, Estrogen/ or (((oestrogen or estrogen) adj3 receptor?) or ERalpha$ or ERbeta or ER-positive or "luminal a" or "luminal b" or (luminal adj2 subtype$) or hormone receptor? or HR positive or ((progesterone or progestin) adj3 receptor?) or PR positive).tw,kf,kw,ot. | 319054 |
| 5 | 1 and 4 [HR BREAST CANCER] | 159843 |
| 6 | exp Breast Neoplasms/sc or exp Breast Carcinoma In Situ/sc or (((advanced or metastatic$ or metastas$ or progressive or secondary or terminal) adj3 ((breast$1 or mamma or mammary) adj3 (adenocarcinoma$ or cancer$ or carcinoma$ or neoplasm$ or tumour$ or tumor$))) or mBC or ((advanced or metastatic$ or metastas$ or progressive or secondary or terminal) adj3 (ductal or intraductal or intra-ductal) adj2 (carcinoma? or hyperplasia?))).tw,kf,kw,ot. [SECONDARY/METASTATIC BREAST CANCER] | 148784 |
| 7 | exp neoplasm metastasis/ or Neoplasm Recurrence, Local/ or ((meta adj sta$) or metastas$ or metastatic$ or recur$ or secondar$ or relaps$ or advance$ or inoperab$ or disseminat$ or spread or migration or lethal$ or incurable or noncurable or non-curable or uncurable or progressive or terminal or invasive$ or aggressive$ or (late? adj2 stage$) or ((stage? or grade? or type?) adj2 (3a$ or 3b$ or 3c$ or III$ or 4a$ or 4b$ or IV$)) or "stage 3" or "stage 4" or met or mets or abc or mbc or m-bc or N1 or N2? or N3? or pN1? or pN2? or pN3?).tw,kf,kw,ot. [METASTASIS] | 13228363 |
| 8 | 1 and 7 [METASTATIC BREAST CANCER] | 574560 |
| 9 | or/6,8 [METASTATIC BREAST CANCER - All terms] | 589325 |
| 10 | (3 and 5) or 9 [BREAST CANCERS OF INTEREST] | 603311 |
| 11 | Salvage Therapy/ or Retreatment/ or (((salvage or "add on") adj2 (therap$ or treatment$ or regime$)) or pretreat$ or pre-treat$ or previously-treat$ or re-treat$ or retreat$ or ((prior or previous$ or subsequent or post or expos$ or fail$) adj2 ("Cyclin-Dependent Kinase 4$" or CDKi$ or CDK4$ or "CDK 4$" or CDK6$ or "CDK 6$")) or ((prior or previous$ or subsequent or multiple or triple?) adj3 (line? or LOT or LOTs or therap$ or treat$ or regime$ or expos$)) or ((multi-cycl$ or multicycl$ or multiple-cycl$ or additional or multiple) adj2 (treat$ or therap$ or regime$ or expos$ or chemotherap$ or chemo-therap$ or dose$)) or add-on-therap$ or secondline? or double-refractory or double-expos$ or thirdline? or triple-refractory or tri-expos$ or triple-expos$ or fourthline? or quadruple-refractory or fifthline? or penta-refractory or penta-expos$ or ((double or two or second or 2nd or "two or more" or "2 prior" or double-class or triple or third or 3rd or "three or more" or "3 prior" or "tri-class" or "triple-class" or quadruple or fourth or 4th or "four or more" or "4 prior" or "quadruple-class" or fifth or penta or 5th or "five or more" or "5 prior" or "penta-class") adj2 (line? or LOT or LOTs or therap$ or regime$ or expos$ or class$2 or drug-class$2))).ti,ab,kf,kw. [RE-TREATMENT/MULTIPLE LINES OF TREATMENT] | 1944822 |
| 12 | 10 and 11 | 50552 |
| 13 | (palbociclib or ibrance$2 or pd0332991 or pd-0332991 or pd332991 or pd-332991 or pf-00080665 or pf00080665 or 571190-30-2 or 827022-33-3 or G9ZF61LE7G or W1NYL2IRDR).ti,ab,kf,ot,hw,rn,nm. [PALBOCICLIB TERMS] | 8264 |
| 14 | (ribociclib or kisqali2$ or lee-011? or lee-11? or lee011? or lee11? or 1211441-98-3 or 1374639-75-4 or TK8ERE8P56 or BG7HLX2919).ti,ab,kf,ot,hw,rn,nm. [RIBOCICLIB TERMS] | 3369 |
| 15 | (abemaciclib or bemaciclib$2 or ly-2835210 or ly-2835219 or ly2835210 or ly2835219 or verzenio$3 or 1231929-97-7 or 1231930-82-7 or 60UAB198HK or KKT462Q807).ti,ab,kf,ot,hw,rn,nm. [ABEMACICLIB TERMS] | 3289 |
| 16 | (dalpiciclib or shr-6390 or shr6390 or 1637781-04-4 or 24DCK9FQ92 or 5ZHA5P4PFX).ti,ab,kf,ot,hw,rn,nm. [DALPICICLIB TERMS] | 82 |
| 17 | (trilaciclib or g1t-28 or g1t-28-1 or g1t28 or g1t28-1 or g1t281 or 1374743-00-6 or 1977495-97-8 or U6072DO9XG or 4BX07W725T).ti,ab,kf,ot,hw,rn,nm. [TRILACICLIB TERMS] | 230 |
| 18 | Cyclin-Dependent Kinase Inhibitor Proteins/ or Cyclin-Dependent Kinases/ or Cyclin-Dependent Kinase 4/ or Cyclin-Dependent Kinase 6/ or (cyclin-dependent kinase inhibitor? or CDKI or CDKIs or CKI Protein? or (CIP-KIP adj2 protein?) or Cyclin-Dependent Kinase 4 or Cyclin-Dependent Kinase 6 or CDK4* or CDK 4* or CDK6* or CDK 6* or Cell Division Protein Kinase 4 or PSK-J3 Kinase or PSKJ3 Kinase or p34PSK-J3 Kinase or p34PSKJ3 Kinase or Cell Division Protein Kinase 6 or PLSTIRE Protein).tw,kw,kf. [CDK4/6i TERMS] | 69625 |
| 19 | or/13-18 [ALL DRUG INTERVENTIONS] | 74287 |
| 20 | 12 and 19 | 2895 |
| 21 | Comparative studies/ or Follow-up studies/ or Time factors/ or (preoperat$ or pre operat$).mp. or (chang$ or evaluat$ or reviewed or prospective$ or retrospective$ or baseline or cohort or case series).tw. [OBSERVATIONAL STUDIES – MEDLINE Filter – max specificity, Fraser, 2006] | 25701542 |
| 22 | exp cohort studies/ or controlled before-after studies/ or interrupted time series analysis/ or historically controlled study/ or case-control studies/ or cross-sectional studies/ or comparative study/ or observational study/ or (((cohort or concurrent or non-concurrent or incidence or follow-up or followup or longitudinal or prospective or retrospective or quasi-experiment$ or pretest or posttest or pre-test or post-test or "before after" or "CBA stud$" or "ITS stud$" or (historical$ adj2 control$) or case-control or case-comparison or case-compeer or case-referrent or case-base or cross-sectional or prevalence) adj3 (stud$ or design?)) or real-world or realworld or RWE or regist$ or (interrupted adj2 time adj2 series)).tw,kf. [RWE STUDIES AND ADDITIONAL TERMS TO SUPPLEMENT FILTERS] | 10474153 |
| 23 | 21 or 22 [OBSERVATIONAL & RWE STUDIES] | 27605837 |
| 24 | 20 and 23 | 2177 |
| 25 | exp Infant/ not exp Adult/ | 1789325 |
| 26 | exp Child/ not exp Adult/ | 3557431 |
| 27 | Adolescent/ not exp Adult/ | 1344196 |
| 28 | 24 not (25 or 26 or 27) [INFANT-, CHILD-, ADOLESCENT-ONLY REMOVED] | 2174 |
| 29 | exp Animals/ not Humans/ | 16585107 |
| 30 | (address or autobiography or bibliography or biography or comment or dictionary or directory or editorial or "expression of concern" or festschrift or historical article or interactive tutorial or lecture or legal case or legislation or news or newspaper article or patient education handout or personal narrative or portrait or video-audio media or webcast or (letter not (letter and randomized controlled trial))).pt. [Opinion publications - MEDLINE] | 4781189 |
| 31 | 28 not (29 or 30) [OPINION PUBLICATIONS & ANIMAL-ONLY REMOVED] | 2061 |
| 32 | 31 use ppez [MEDLINE RECORDS] | 346 |
| 33 | exp breast cancer/ or (((breast$ or mamma or mammary) adj3 (adenocarcinoma$ or cancer$ or carcinoma$ or neoplasm$ or tumour$ or tumor$ or malignan$)) or ((ductal or duct or intraductal or intra-ductal) adj1 (carcinoma? or hyper-plasia? or hyperplasia?)) or (lobul$ carcinoma? adj2 "in situ") or (paget$ and (areola? or breast$ or mammary or nipple$)) or ((IBC or DCIS or LCIS) and (breast$ or mamma or mammary))).tw,kf,kw,ot. [BREAST CANCER] | 1216212 |
| 34 | epidermal growth factor receptor 2/ or (ErbB2 or "ErbB 2" or HER2$ or "HER 2$" or "c-ErbB2" or "C-ErbB 2" or ((oncoprotein$ or onco-protein$ or protein$ or receptor$) adj1 (neu or neuregulin)) or CD340 or "p185(c-neu)" or p185erbB or "neu protooncogene" or "neu proto-oncogene" or NGL or "metastatic lymph node gene 19" or MLN19 or MLN 19 or "epidermal growth factor receptor 2" or EGFR2 or "EGFR 2" or "HR+/HER2+" or "HR+/HER 2+" or "ER+/HER2+" or "ER+/HER 2+" or "HR+/HER2-" or "HR+/HER 2-" or "ER+/HER2-" or "ER+/HER 2-").tw,kf,kw,ot. [Erb-b2 RECEPTOR TERMS] | 178146 |
| 35 | 33 and 34 [HER2 BREAST CANCER] | 125980 |
| 36 | exp estrogen receptor/ or exp estrogen receptor positive breast cancer/ or progesterone receptor positive breast cancer/ or (((oestrogen or estrogen) adj3 receptor?) or ERalpha$ or ERbeta or ER-positive or "luminal a" or "luminal b" or (luminal adj2 subtype$) or hormone receptor? or HR positive or ((progesterone or progestin) adj3 receptor?) or PR positive).tw,kf,kw,ot. [HR BREAST CANCER] | 321458 |
| 37 | 33 and 36 [HR BREAST CANCER] | 161786 |
| 38 | exp Breast Neoplasms/sc or exp Breast Carcinoma In Situ/sc or (((advanced or metastatic$ or metastas$ or progressive or secondary or terminal) adj3 ((breast$1 or mamma or mammary) adj3 (adenocarcinoma$ or cancer$ or carcinoma$ or neoplasm$ or tumour$ or tumor$))) or mBC or ((advanced or metastatic$ or metastas$ or progressive or secondary or terminal) adj3 (ductal or intraductal or intra-ductal) adj2 (carcinoma? or hyperplasia?))).tw,kf,kw,ot. [SECONDARY/METASTATIC BREAST CANCER] | 148784 |
| 39 | exp metastasis/ or exp cancer recurrence/ or exp advanced cancer/ or ((meta adj sta$) or metastas$ or metastatic$ or recur$ or secondar$ or relaps$ or advance$ or inoperab$ or disseminat$ or spread or migration or lethal$ or incurable or noncurable or non-curable or uncurable or progressive or terminal or invasive$ or aggressive$ or (late? adj2 stage$) or ((stage? or grade? or type?) adj2 (3a$ or 3b$ or 3c$ or III$ or 4a$ or 4b$ or IV$)) or "stage 3" or "stage 4" or met or mets or abc or mbc or m-bc or N1 or N2? or N3? or pN1? or pN2? or pN3?).tw,kf,kw,ot. [METASTASIS] | 13233528 |
| 40 | 33 and 39 [METASTATIC BREAST CANCER] | 572472 |
| 41 | or/38,40 [METASTATIC BREAST CANCER - All terms] | 587305 |
| 42 | (35 and 37) or 41 [BREAST CANCERS OF INTEREST] | 599846 |
| 43 | salvage therapy/ or retreatment/ or multiple cycle treatment/ or "add on therapy"/ or (((salvage or "add on") adj2 (therap$ or treatment$ or regime$)) or pretreat$ or pre-treat$ or previously-treat$ or re-treat$ or retreat$ or ((prior or previous$ or subsequent or post or expos$ or fail$) adj2 ("Cyclin-Dependent Kinase 4$" or CDKi$ or CDK4$ or "CDK 4$" or CDK6$ or "CDK 6$")) or ((prior or previous$ or subsequent or multiple or triple?) adj3 (line? or LOT or LOTs or therap$ or treat$ or regime$ or expos$)) or ((multi-cycl$ or multicycl$ or multiple-cycl$ or additional or multiple) adj2 (treat$ or therap$ or regime$ or expos$ or chemotherap$ or chemo-therap$ or dose$)) or add-on-therap$ or secondline? or double-refractory or double-expos$ or thirdline? or triple-refractory or tri-expos$ or triple-expos$ or fourthline? or quadruple-refractory or fifthline? or penta-refractory or penta-expos$ or ((double or two or second or 2nd or "two or more" or "2 prior" or double-class or triple or third or 3rd or "three or more" or "3 prior" or "tri-class" or "triple-class" or quadruple or fourth or 4th or "four or more" or "4 prior" or "quadruple-class" or fifth or penta or 5th or "five or more" or "5 prior" or "penta-class") adj2 (line? or LOT or LOTs or therap$ or regime$ or expos$ or class$2 or drug-class$2))).ti,ab,kf,kw. [RE-TREATMENT/MULTIPLE LINES OF TREATMENT] | 2039569 |
| 44 | 42 and 43 | 58801 |
| 45 | palbociclib/ or (palbociclib or ibrance$2 or pd0332991 or pd-0332991 or pd332991 or pd-332991 or pf-00080665 or pf00080665 or 571190-30-2 or 827022-33-3 or G9ZF61LE7G or W1NYL2IRDR).ti,ab,kf,ot,rn,dq. [PALBOCICLIB TERMS] | 8261 |
| 46 | ribociclib/ or (ribociclib or kisqali2$ or lee-011? or lee-11? or lee011? or lee11? or 1211441-98-3 or 1374639-75-4 or TK8ERE8P56 or BG7HLX2919).ti,ab,kf,ot,hw,rn,nm. [RIBOCICLIB TERMS] | 3369 |
| 47 | abemaciclib/ or (abemaciclib or bemaciclib$2 or ly-2835210 or ly-2835219 or ly2835210 or ly2835219 or verzenio$3 or 1231929-97-7 or 1231930-82-7 or 60UAB198HK or KKT462Q807).ti,ab,kf,ot,rn,dq. [ABEMACICLIB TERMS] | 3289 |
| 48 | dalpiciclib/ or (dalpiciclib or shr-6390 or shr6390 or 1637781-04-4 or 24DCK9FQ92 or 5ZHA5P4PFX).ti,ab,kf,ot,rn,dq. [DALPICICLIB TERMS] | 82 |
| 49 | trilaciclib/ or (trilaciclib or g1t-28 or g1t-28-1 or g1t28 or g1t28-1 or g1t281 or 1374743-00-6 or 1977495-97-8 or U6072DO9XG or 4BX07W725T).ti,ab,kf,ot,rn,dq. [TRILACICLIB TERMS] | 230 |
| 50 | cyclin dependent kinase inhibitor/ or cyclin dependent kinase/ or (cyclin-dependent kinase inhibitor? or CDKI or CDKIs or CKI Protein? or (CIP-KIP adj2 protein?) or Cyclin-Dependent Kinase 4 or Cyclin-Dependent Kinase 6 or CDK4* or CDK 4* or CDK6* or CDK 6* or Cell Division Protein Kinase 4 or PSK-J3 Kinase or PSKJ3 Kinase or p34PSK-J3 Kinase or p34PSKJ3 Kinase or Cell Division Protein Kinase 6 or PLSTIRE Protein).tw,kw,kf. [CDK4/6i TERMS] | 65192 |
| 51 | or/45-50 [ALL DRUG INTERVENTIONS] | 70102 |
| 52 | 44 and 51 | 3101 |
| 53 | Controlled study/ or Treatment outcome/ or Major clinical study/ or Clinical trial/ or (chang$ or evaluat$ or reviewed or baseline or (compare$ or compara$)).tw. [OBSERVATIONAL STUDIES – Embase Filter – max specificity, Fraser, 2006] | 32949917 |
| 54 | exp cohort analysis/ or exp case control study/ or controlled clinical trial/ or pretest posttest control group design/ or static group comparison/ or retrospective study/ or longitudinal study/ or intervention study/ or family study/ or case study/ or time series analysis/ or cross-sectional study/ or comparative study/ or observational study/ or quasi experimental study/ or (((cohort or concurrent or non-concurrent or incidence or follow-up or followup or longitudinal or prospective or retrospective or quasi-experiment$ or pretest or posttest or pre-test or post-test or "before after" or "CBA stud$" or "ITS stud$" or (historical$ adj2 control$) or case-control or case-comparison or case-compeer or case-referrent or case-base or cross-sectional or prevalence) adj3 (stud$ or design?)) or real-world or realworld or RWE or regist$ or (interrupted adj2 time adj2 series)).tw,kw,kf. [RWE STUDIES AND ADDITIONAL TERMS TO SUPPLEMENT FILTERS] | 13960762 |
| 55 | 53 or 54 [OBSERVATIONAL & RWE STUDIES] | 37789608 |
| 56 | 52 and 55 | 2626 |
| 57 | adolescent/ not exp adult/ | 1344196 |
| 58 | child/ not exp adult/ | 2547815 |
| 59 | infant/ not exp adult/ | 1131423 |
| 60 | fetus/ not exp adult/ | 237913 |
| 61 | 56 not (57 or 58 or 59 or 60) [FETUS-, INFANT-, CHILD-, ADOLESCENT-ONLY REMOVED] | 2623 |
| 62 | exp animal/ or exp animal experimentation/ or exp animal model/ or exp animal experiment/ or nonhuman/ or exp vertebrate/ | 58159098 |
| 63 | exp human/ or exp human experimentation/ or exp human experiment/ | 46026506 |
| 64 | 62 not 63 | 12134436 |
| 65 | 61 not 64 [ANIMAL-ONLY REMOVED] | 2582 |
| 66 | (editorial or letter or note or short survey or tombstone).pt. [OPINION PIECES REMOVE - Embase] | 5142396 |
| 67 | 65 not 66 | 2572 |
| 68 | conference abstract.pt. | 4621722 |
| 69 | 67 not 68 [CONFERENCE ABSTRACTS REMOVED] | 1510 |
| 70 | 67 and 68 | 1062 |
| 71 | limit 70 to yr="2019-current" | 738 |
| 72 | 69 or 71 [MOST RECENT 3 YRS CONFERENCE ABSTRACTS RETAINED] | 2248 |
| 73 | 72 use oemezd [EMBASE RECORDS] | 1397 |
| 74 | exp Breast Neoplasms/ or exp Breast Carcinoma In Situ/ or (((breast$ or mamma or mammary) adj3 (adenocarcinoma$ or cancer$ or carcinoma$ or neoplasm$ or tumour$ or tumor$ or malignan$)) or ((ductal or duct or intraductal or intra-ductal) adj1 (carcinoma? or hyper-plasia? or hyperplasia?)) or (lobul$ carcinoma? adj2 "in situ") or (paget$ and (areola? or breast$ or mammary or nipple$)) or ((IBC or DCIS or LCIS) and (breast$ or mamma or mammary))).tw,kw. [BREAST CANCER] | 1233988 |
| 75 | Receptor, ErbB-2/ or ErbB Receptors/ or (ErbB2 or "ErbB 2" or HER2$ or "HER 2$" or "c-ErbB2" or "C-ErbB 2" or ((oncoprotein$ or onco-protein$ or protein$ or receptor$) adj1 (neu or neuregulin)) or CD340 or "p185(c-neu)" or p185erbB or "neu protooncogene" or "neu proto-oncogene" or NGL or "metastatic lymph node gene 19" or MLN19 or MLN 19 or "epidermal growth factor receptor 2" or EGFR2 or "EGFR 2" or "HR+/HER2+" or "HR+/HER 2+" or "ER+/HER2+" or "ER+/HER 2+" or "HR+/HER2-" or "HR+/HER 2-" or "ER+/HER2-" or "ER+/HER 2-").tw,kw. [Erb-b2 RECEPTOR TERMS] | 307626 |
| 76 | 74 and 75 [HER2 BREAST CANCER] | 137365 |
| 77 | exp Receptors, Estrogen/ or (((oestrogen or estrogen) adj3 receptor?) or ERalpha$ or ERbeta or ER-positive or "luminal a" or "luminal b" or (luminal adj2 subtype$) or hormone receptor? or HR positive or ((progesterone or progestin) adj3 receptor?) or PR positive).tw,kw. [HR BREAST CANCER] | 315332 |
| 78 | 74 and 77 [HR BREAST CANCER] | 158826 |
| 79 | exp Breast Neoplasms/sc or exp Breast Carcinoma In Situ/sc or (((advanced or metastatic$ or metastas$ or progressive or secondary or terminal) adj3 ((breast$1 or mamma or mammary) adj3 (adenocarcinoma$ or cancer$ or carcinoma$ or neoplasm$ or tumour$ or tumor$))) or mBC or ((advanced or metastatic$ or metastas$ or progressive or secondary or terminal) adj3 (ductal or intraductal or intra-ductal) adj2 (carcinoma? or hyperplasia?))).tw,kw. [SECONDARY/METASTATIC BREAST CANCER] | 147922 |
| 80 | exp neoplasm metastasis/ or Neoplasm Recurrence, Local/ or ((meta adj sta$) or metastas$ or metastatic$ or recur$ or secondar$ or relaps$ or advance$ or inoperab$ or disseminat$ or spread or migration or lethal$ or incurable or noncurable or non-curable or uncurable or progressive or terminal or invasive$ or aggressive$ or (late? adj2 stage$) or ((stage? or grade? or type?) adj2 (3a$ or 3b$ or 3c$ or III$ or 4a$ or 4b$ or IV$)) or "stage 3" or "stage 4" or met or mets or abc or mbc or m-bc or N1 or N2? or N3? or pN1? or pN2? or pN3?).tw,kw. [METASTASIS] | 13164512 |
| 81 | 74 and 80 [METASTATIC BREAST CANCER] | 571658 |
| 82 | or/79,81 [METASTATIC BREAST CANCER] | 586330 |
| 83 | (76 and 78) or 82 [BREAST CANCERS OF INTEREST] | 600279 |
| 84 | Salvage Therapy/ or Retreatment/ or (((salvage or "add on") adj2 (therap$ or treatment$ or regime$)) or pretreat$ or pre-treat$ or previously-treat$ or re-treat$ or retreat$ or ((prior or previous$ or subsequent or post or expos$ or fail$) adj2 ("Cyclin-Dependent Kinase 4$" or CDKi$ or CDK4$ or "CDK 4$" or CDK6$ or "CDK 6$")) or ((prior or previous$ or subsequent or multiple or triple?) adj3 (line? or LOT or LOTs or therap$ or treat$ or regime$ or expos$)) or ((multi-cycl$ or multicycl$ or multiple-cycl$ or additional or multiple) adj2 (treat$ or therap$ or regime$ or expos$ or chemotherap$ or chemo-therap$ or dose$)) or add-on-therap$ or secondline? or double-refractory or double-expos$ or thirdline? or triple-refractory or tri-expos$ or triple-expos$ or fourthline? or quadruple-refractory or fifthline? or penta-refractory or penta-expos$ or ((double or two or second or 2nd or "two or more" or "2 prior" or double-class or triple or third or 3rd or "three or more" or "3 prior" or "tri-class" or "triple-class" or quadruple or fourth or 4th or "four or more" or "4 prior" or "quadruple-class" or fifth or penta or 5th or "five or more" or "5 prior" or "penta-class") adj2 (line? or LOT or LOTs or therap$ or regime$ or expos$ or class$2 or drug-class$2))).ti,ab,kw. [RE-TREATMENT/MULTIPLE LINES OF TREATMENT] | 1942167 |
| 85 | 83 and 84 | 50378 |
| 86 | (palbociclib or ibrance$2 or pd0332991 or pd-0332991 or pd332991 or pd-332991 or pf-00080665 or pf00080665 or 571190-30-2 or 827022-33-3 or G9ZF61LE7G or W1NYL2IRDR).ti,ab,kw. [PALBOCICLIB TERMS] | 5707 |
| 87 | (ribociclib or kisqali2$ or lee-011? or lee-11? or lee011? or lee11? or 1211441-98-3 or 1374639-75-4 or TK8ERE8P56 or BG7HLX2919).ti,ab,kw. [RIBOCICLIB TERMS] | 2251 |
| 88 | (abemaciclib or bemaciclib$2 or ly-2835210 or ly-2835219 or ly2835210 or ly2835219 or verzenio$3 or 1231929-97-7 or 1231930-82-7 or 60UAB198HK or KKT462Q807).ti,ab,kw. [ABEMACICLIB TERMS] | 2177 |
| 89 | (dalpiciclib or shr-6390 or shr6390 or 1637781-04-4 or 24DCK9FQ92 or 5ZHA5P4PFX).ti,ab,kw. [DALPICICLIB TERMS] | 56 |
| 90 | (trilaciclib or g1t-28 or g1t-28-1 or g1t28 or g1t28-1 or g1t281 or 1374743-00-6 or 1977495-97-8 or U6072DO9XG or 4BX07W725T).ti,ab,kw. [TRILACICLIB TERMS] | 177 |
| 91 | Cyclin-Dependent Kinase Inhibitor Proteins/ or Cyclin-Dependent Kinases/ or Cyclin-Dependent Kinase 4/ or Cyclin-Dependent Kinase 6/ or (cyclin-dependent kinase inhibitor? or CDKI or CDKIs or CKI Protein? or (CIP-KIP adj2 protein?) or Cyclin-Dependent Kinase 4 or Cyclin-Dependent Kinase 6 or CDK4* or CDK 4* or CDK6* or CDK 6* or Cell Division Protein Kinase 4 or PSK-J3 Kinase or PSKJ3 Kinase or p34PSK-J3 Kinase or p34PSKJ3 Kinase or Cell Division Protein Kinase 6 or PLSTIRE Protein).tw,kw. [CDK4/6i TERMS] | 69376 |
| 92 | or/86-91 [ALL DRUG INTERVENTIONS] | 71906 |
| 93 | 85 and 92 | 2796 |
| 94 | Comparative studies/ or Follow-up studies/ or Time factors/ or (preoperat$ or pre operat$).mp. or (chang$ or evaluat$ or reviewed or prospective$ or retrospective$ or baseline or cohort or case series).tw. [OBSERVATIONAL STUDIES – MEDLINE/CENTRAL Filter – max specificity, Fraser, 2006] | 25701542 |
| 95 | exp Cohort Studies/ or Controlled Before-After Studies/ or Interrupted Time Series Analysis/ or Historically Controlled Study/ or Case-Control Studies/ or Cross-Sectional Studies/ or Comparative Study/ or Observational Study/ or (((cohort or concurrent or non-concurrent or incidence or follow-up or followup or longitudinal or prospective or retrospective or quasi-experiment$ or pretest or posttest or pre-test or post-test or "before after" or "CBA stud$" or "ITS stud$" or (historical$ adj2 control$) or case-control or case-comparison or case-compeer or case-referrent or case-base or cross-sectional or prevalence) adj3 (stud$ or design?)) or real-world or realworld or RWE or regist$ or (interrupted adj2 time adj2 series)).tw,kw. [RWE STUDIES AND ADDITIONAL TERMS TO SUPPLEMENT OBSERVATIONAL, RWE FILTERS] | 10509849 |
| 96 | 94 or 95 [OBSERVATIONAL & RWE STUDIES] | 27614421 |
| 97 | 85 and 96 | 32966 |
| 98 | exp Infant/ not exp Adult/ | 1789325 |
| 99 | exp Child/ not exp Adult/ | 3557431 |
| 100 | Adolescent/ not exp Adult/ | 1344196 |
| 101 | (editorial or letter or comment or note).pt. [OPINION PIECES REMOVE - CENTRAL] | 5057789 |
| 102 | 97 not (98 or 99 or 100 or 101) [INFANT-, CHILD-, ADOLESCENT-ONLY & OPINION PIECES REMOVED] | 32804 |
| 103 | conference proceeding.pt. | 214308 |
| 104 | 102 not 103 [CONFERENCE ABSTRACTS REMOVED] | 31468 |
| 105 | 102 and 103 [CONFERENCE ABSTRACTS ONLY] | 1336 |
| 106 | limit 105 to yr="2019 -Current" | 563 |
| 107 | 104 or 106 [MOST RECENT 3 YRS CONFERENCE ABSTRACTS RETAINED] | 32031 |
| 108 | 107 use cctr [CENTRAL RECORDS] | 3057 |
| 109 | (((breast$ or mamma or mammary) adj3 (adenocarcinoma$ or cancer$ or carcinoma$ or neoplasm$ or tumour$ or tumor$ or malignan$)) or ((ductal or duct or intraductal or intra-ductal) adj1 (carcinoma? or hyper-plasia? or hyperplasia?)) or (lobul$ carcinoma? adj2 "in situ") or (paget$ and (areola? or breast$ or mammary or nipple$)) or ((IBC or DCIS or LCIS) and (breast$ or mamma or mammary))).tw,kw. [BREAST CANCER] | 1033223 |
| 110 | (ErbB2 or "ErbB 2" or HER2$ or "HER 2$" or "c-ErbB2" or "C-ErbB 2" or ((oncoprotein$ or onco-protein$ or protein$ or receptor$) adj1 (neu or neuregulin)) or CD340 or "p185(c-neu)" or p185erbB or "neu protooncogene" or "neu proto-oncogene" or NGL or "metastatic lymph node gene 19" or MLN19 or MLN 19 or "epidermal growth factor receptor 2" or EGFR2 or "EGFR 2" or "HR+/HER2+" or "HR+/HER 2+" or "ER+/HER2+" or "ER+/HER 2+" or "HR+/HER2-" or "HR+/HER 2-" or "ER+/HER2-" or "ER+/HER 2-").tw,kw. [Erb-b2 RECEPTOR TERMS] | 158375 |
| 111 | 109 and 110 [HER2 BREAST CANCER] | 111639 |
| 112 | (((oestrogen or estrogen) adj3 receptor?) or ERalpha$ or ERbeta or ER-positive or "luminal a" or "luminal b" or (luminal adj2 subtype$) or hormone receptor? or HR positive or ((progesterone or progestin) adj3 receptor?) or PR positive).tw,kw. [HR BREAST CANCER] | 275640 |
| 113 | 109 and 112 [HR BREAST CANCER] | 133900 |
| 114 | (((advanced or metastatic$ or metastas$ or progressive or secondary or terminal) adj3 ((breast$1 or mamma or mammary) adj3 (adenocarcinoma$ or cancer$ or carcinoma$ or neoplasm$ or tumour$ or tumor$))) or mBC or ((advanced or metastatic$ or metastas$ or progressive or secondary or terminal) adj3 (ductal or intraductal or intra-ductal) adj2 (carcinoma? or hyperplasia?))).tw,kw. [SECONDARY/METASTATIC BREAST CANCER] | 144867 |
| 115 | ((meta adj sta$) or metastas$ or metastatic$ or recur$ or secondar$ or relaps$ or advance$ or inoperab$ or disseminat$ or spread or migration or lethal$ or incurable or noncurable or non-curable or uncurable or progressive or terminal or invasive$ or aggressive$ or (late? adj2 stage$) or ((stage? or grade? or type?) adj2 (3a$ or 3b$ or 3c$ or III$ or 4a$ or 4b$ or IV$)) or "stage 3" or "stage 4" or met or mets or abc or mbc or m-bc or N1 or N2? or N3? or pN1? or pN2? or pN3?).tw,kw. [METASTASIS] | 12991643 |
| 116 | 109 and 115 [METASTATIC BREAST CANCER] | 495840 |
| 117 | or/114,116 [METASTATIC BREAST CANCER - All terms] | 510308 |
| 118 | (111 and 113) or 117 [BREAST CANCERS OF INTEREST] | 520617 |
| 119 | (((salvage or "add on") adj2 (therap$ or treatment$ or regime$)) or pretreat$ or pre-treat$ or previously-treat$ or re-treat$ or retreat$ or ((prior or previous$ or subsequent or post or expos$ or fail$) adj2 ("Cyclin-Dependent Kinase 4$" or CDKi$ or CDK4$ or "CDK 4$" or CDK6$ or "CDK 6$")) or ((prior or previous$ or subsequent or multiple or triple?) adj3 (line? or LOT or LOTs or therap$ or treat$ or regime$ or expos$)) or ((multi-cycl$ or multicycl$ or multiple-cycl$ or additional or multiple) adj2 (treat$ or therap$ or regime$ or expos$ or chemotherap$ or chemo-therap$ or dose$)) or add-on-therap$ or secondline? or double-refractory or double-expos$ or thirdline? or triple-refractory or tri-expos$ or triple-expos$ or fourthline? or quadruple-refractory or fifthline? or penta-refractory or penta-expos$ or ((double or two or second or 2nd or "two or more" or "2 prior" or double-class or triple or third or 3rd or "three or more" or "3 prior" or "tri-class" or "triple-class" or quadruple or fourth or 4th or "four or more" or "4 prior" or "quadruple-class" or fifth or penta or 5th or "five or more" or "5 prior" or "penta-class") adj2 (line? or LOT or LOTs or therap$ or regime$ or expos$ or class$2 or drug-class$2))).ti,ab,kw. [RE-TREATMENT/MULTIPLE LINES OF TREATMENT] | 1908229 |
| 120 | 118 and 119 | 46774 |
| 121 | (palbociclib or ibrance$2 or pd0332991 or pd-0332991 or pd332991 or pd-332991 or pf-00080665 or pf00080665 or 571190-30-2 or 827022-33-3 or G9ZF61LE7G or W1NYL2IRDR).ti,ab,kw. [PALBOCICLIB TERMS] | 5707 |
| 122 | (ribociclib or kisqali2$ or lee-011? or lee-11? or lee011? or lee11? or 1211441-98-3 or 1374639-75-4 or TK8ERE8P56 or BG7HLX2919).ti,ab,kw. [RIBOCICLIB TERMS] | 2251 |
| 123 | (abemaciclib or bemaciclib$2 or ly-2835210 or ly-2835219 or ly2835210 or ly2835219 or verzenio$3 or 1231929-97-7 or 1231930-82-7 or 60UAB198HK or KKT462Q807).ti,ab,kw. [ABEMACICLIB TERMS] | 2177 |
| 124 | (dalpiciclib or shr-6390 or shr6390 or 1637781-04-4 or 24DCK9FQ92 or 5ZHA5P4PFX).ti,ab,kw. [DALPICICLIB TERMS] | 56 |
| 125 | (trilaciclib or g1t-28 or g1t-28-1 or g1t28 or g1t28-1 or g1t281 or 1374743-00-6 or 1977495-97-8 or U6072DO9XG or 4BX07W725T).ti,ab,kw. [TRILACICLIB TERMS] | 177 |
| 126 | (cyclin-dependent kinase inhibitor? or CDKI or CDKIs or CKI Protein? or (CIP-KIP adj2 protein?) or Cyclin-Dependent Kinase 4 or Cyclin-Dependent Kinase 6 or CDK4* or CDK 4* or CDK6* or CDK 6* or Cell Division Protein Kinase 4 or PSK-J3 Kinase or PSKJ3 Kinase or p34PSK-J3 Kinase or p34PSKJ3 Kinase or Cell Division Protein Kinase 6 or PLSTIRE Protein).tw,kw. [CDK4/6i TERMS] | 45060 |
| 127 | or/121-126 [ALL DRUG INTERVENTIONS] | 47725 |
| 128 | 120 use coch [CDSR RECORDS] | 45 |
| 129 | 32 or 73 or 108 or 128 | 4845 |
| 130 | limit 129 to yr="2015 -Current" | 3377 |
| 131 | limit 130 to yr="2021 -Current" | 1365 |
| 132 | remove duplicates from 131 | 1159 |
| 133 | 130 not 131 | 2012 |
| 134 | remove duplicates from 133 | 1725 |
| **135** | **132 or 134 [ALL DATABASES - DEDUPLICATED]** | **2884** |
| 136 | 135 use ppez [MEDLINE RECORDS] | 29 |
| 137 | 135 use oemezd [EMBASE RECORDS] | 1325 |
| 138 | 135 use cctr [CENTRAL RECORDS] | 1510 |
| 139 | 135 use coch [CDSR RECORDS] | 20 |

EndNote deduplication process: 125 duplicates were removed; **final recall: 2759 hits**

**Table S1**. List of studies excluded during full-text screening

| **Author** | **Title** | **Year** | **Reason for Exclusion** |
| --- | --- | --- | --- |
| Agrawal, C., Goyal, P., Agarwal, A., Tripathi, R., Dodagoudar, C., Baghmar, S., Sharma, A., Batra, U., Talwar, V., Goyal, S., Kumar, R., Doval, D. C. | Multicentric real world evidence with palbociclib in hormone positive HER2 negative metastatic breast cancer in Indian population | 2021 | Population - mixed patient population or unclear whether population has failed CDK4/6i |
| Al-Foheidi, M. H., Albeshri, A. M., Moamenkahan, S. N., Abdullah, A. M., Abualola, M. S., Alharbi, M. H., Refa, A. A., Bayer, A. M., Shaheen, A. Y., Aga, S. S., Khan, M. A., Al-Mansour, M. M., Ibrahim, E. M. | Combination of palbociclib with adjuvant endocrine therapy for treatment of hormone receptor-positive and human epidermal growth factor receptor 2-negative metastatic breast cancer: An experience at two cancer centers in Saudi Arabia | 2022 | Population - all other reasons |
| Amaro, C. P., Batra, A., Lupichuk, S. | First-line treatment with a cyclin-dependent kinase 4/6 inhibitor combined with an aromatase inhibitor for hormone receptor positive, human epidermal growth factor receptor-2 negative metastatic breast cancer: Population-based outcomes for patients treated in Alberta, Canada | 2021 | Duplicate |
| Amaro, C., Batra, A., Lupichuk, S. | First-line treatment with a cyclin-dependent kinase 4/6 inhibitor plus an aromatase inhibitor for metastatic breast cancer in Alberta | 2021 | Duplicate |
| Anonymous | Capecitabine efficacy after progression on endocrine treatment and cycline-dependant-kinase 4/6 inhibitor combination in metastatic hormone-receptor positive breast cancer | 2022 | Population - failed CDK4/6i but not in 1L |
| Araujo, A., Costa, M. I., Dimas, F., Carreteiro, C., Andreozzi, V. | POSB5 Real-World Patterns of Treatment and Dose Modification with CDKI 4/6 for Advanced Breast Cancer in Portugal | 2022 | Population - mixed patient population or unclear whether population has failed CDK4/6i |
| Balmana, J., Fasching, P. A., Delaloge, S., Park, Y. H., Eisen, A., Bourgeois, H., Kemp, Z., Jankowski, T., Sohn, J., Aksoy, S., Timcheva, C. V., Park-Simon, T. W., Anton Torres, A., John, E., Baria, K., Walker, G., Gelmon, K. A. | Clinical effectiveness and safety of olaparib in BRCA-mutated, HER2-negative metastatic breast cancer in a real-world setting: Phase IIIb LUCY final analysis | 2022 | Population - mixed patient population or unclear whether population has failed CDK4/6i |
| Ban, M. | Optimal treatment of avanced luminal breast cancer | 2020 | Study design |
| Ban, M., Mise, B. P., Majic, A., Drazic, I., Vrdoljak, E. | Efficacy and safety of palbociclib in heavily pretreated patients with HR+/HER2- metastatic breast cancer | 2018 | Population - mixed patient population or unclear whether population has failed CDK4/6i |
| Basile, D., Gerratana, L., Corvaja, C., Pelizzari, G., Franceschin, G., Bertoli, E., Palmero, L., Zara, D., Alberti, M., Buriolla, S., Da Ros, L., Bonotto, M., Mansutti, M., Spazzapan, S., Cinausero, M., Minisini, A. M., Fasola, G., Puglisi, F. | First- and second-line treatment strategies for hormone-receptor (HR)-positive HER2-negative metastatic breast cancer: A real-world study | 2021 | Population - mixed patient population or unclear whether population has failed CDK4/6i |
| Basile, D., Gerratana, L., Pelizzari, G., Franceschin, G., Buriolla, S., Corvaja, C., Bertoli, E., Palmero, L., Zara, D., Alberti, M., Da Ros, L., Mansutti, M., Spazzapan, S., Bolzonello, S., Bonotto, M., Minisini, A. M., Cinausero, M., Fasola, G. | First-and second-line treatment strategies for hormone-receptor (HR)-positive her2-negative metastatic breast cancer: The PLBC study | 2020 | Duplicate |
| Battisti, N. M. L., Kingston, B., King, J., Denton, A., Waters, S., Sita-Lumsden, A., Rehman, F., Stavraka, C., Kristeleit, H., Sawyer, E., Houghton, D., Davidson, N., Howell, S., Choy, J., Harper, P., Roylance, R., Fharat, R., Mohammed, K., Ring, A., Johnston, S. | Palbociclib and endocrine therapy in fourth line and beyond for hormone receptor-positive HER2-negative advanced breast cancer: The UK compassionate access program experience | 2019 | Population - mixed patient population or unclear whether population has failed CDK4/6i |
| Battisti, N. M. L., Kingston, B., King, J., Denton, A., Waters, S., Sita-Lumsden, A., Rehman, F., Stavraka, C., Kristeleit, H., Sawyer, E., Houghton, D., Davidson, N., Howell, S., Choy, J., Harper, P., Roylance, R., Fharat, R., Mohammed, K., Ring, A., Johnston, S. | Palbociclib and endocrine therapy in heavily pretreated hormone receptor-positive HER2-negative advanced breast cancer: the UK Compassionate Access Programme experience | 2019 | Population - mixed patient population or unclear whether population has failed CDK4/6i |
| Bello, D., Bertucci, A., De La Motte Rouge, T., Blonz, C., Akla, S., Grenier, J., Bailleux, C., Benderra, M. A., Simon, H., Desmoulins, I., Tharin, Z., Renaud, E., Delaloge, S., Bertho, M., Cottu, P. H., Goncalves, A., Bidard, F. C., Lerebours, F. | Alpelisib and fulvestrant efficacy in HR-positive HER2-negative PIK3CA-mutant advanced breast cancer: Data from the French early access program | 2021 | Population - failed CDK4/6i but not in 1L |
| Blum, J. L., Dicristo, C., Gordon, D., Karuturi, M., Oubre, D., Jepsen, E., Cuevas, J., Lakhanpal, S., Zhang, Z., Drucker, M., Wang, Y., Tripathy, D. | Palbociclib (PAL) in male patients (pts) with hormone receptor-positive/human epidermal growth factor receptor 2-negative (HR+/HER2-) advanced breast cancer (ABC): Pt characteristics and treatment (Tx) patterns from the POLARIS study | 2021 | Population - mixed patient population or unclear whether population has failed CDK4/6i |
| Boer, K., Rubovszky, G., Rokszin, G., Abonyi-Toth, Z., Foldesi, C., Dank, M. | Demographic characteristics and treatment patterns among patients receiving palbociclib for hr+/her2- advanced breast cancer: A nationwide real-world experience | 2021 | Population - mixed patient population or unclear whether population has failed CDK4/6i |
| Bottosso, M., Giarratano, T., Barbieri, C., Menichetti, A., Cumerlato, E., Miglietta, F., Genovesi, E., Amato, O., Dieci, M. V., Griguolo, G., Guarneri, V. | Abemaciclib in HR+/HER2- metastatic breast cancer: A real-world experience | 2021 | Population - mixed patient population or unclear whether population has failed CDK4/6i |
| Bouteiller, F., Pulido, M., Brain, E., Paillaud, E., Grosjean, J., Mina, W., Caillet, P., Tassy, L., Soubeyran, P., Rifi, N., Falandry, C., Carola, E. | | 2022 | Duplicate |
| Brain, E., Pulido, M., Paillaud, E., Grosjean, J., Mina, W., Caillet, P., Tassy, L., Soubeyran, P., Bouteiller, F., Rifi, N., Vauthier, J. M., Falandry, C., Carola, E. | Feasibility of palbociclib in women aged 70 and older with resistant and/or pretreated advanced breast cancer in the PALOMAGE study | 2022 | Population - mixed patient population or unclear whether population has failed CDK4/6i |
| Brufsky, A., Mitra, D., Davis, K. L., Nagar, S. P., McRoy, L., Cotter, M. J., Stearns, V. | Treatment Patterns and Outcomes Associated With Palbociclib Plus Letrozole for Postmenopausal Women With HR+/HER2- Advanced Breast Cancer Enrolled in an Expanded Access Program | 2019 | Population - mixed patient population or unclear whether population has failed CDK4/6i |
| Bruno, L., Ostinelli, A., Waisberg, F., Enrico, D., Ponce, C., Rivero, S., Blanco, A., Zarba, M., Loza, M., Fabiano, V., Amat, M., Pombo, M. T., Noro, L., Chacon, M., Colo, F., Chacon, R., Nadal, J., Nervo, A., Costanzo, V. | Cyclin-Dependent Kinase 4/6 Inhibitor Outcomes in Patients With Advanced Breast Cancer Carrying Germline Pathogenic Variants in DNA Repair-Related Genes | 2022 | Population - mixed patient population or unclear whether population has failed CDK4/6i |
| Burattini, E., Astone, A., Satta, F., Pellegrino, A., Todi, F., Zoffoli, M. V., Marchetti, L., Ramundo, M., Ludovisi, E. | Palbociclib: CDK4/6 inhibitors: Efficacy and toxicity evaluation: Our experience in advanced breast cancer | 2019 | Population - mixed patient population or unclear whether population has failed CDK4/6i |
| Caillet, P., Pulido, M., Brain, E., Falandry, C., Desmoulins, I., Ghebriou, D., Soubeyran, P. L., Paillaud, E., Rifi, N., Vauthier, J. M., Tassy, L., Carola, E. | PALOMAGE, a French real-world cohort of elderly women beyond age 70 with advanced breast cancer receiving palbociclib: Baseline characteristics and safety evaluation | 2021 | Population - mixed patient population or unclear whether population has failed CDK4/6i |
| Cantarelli, L., Morales Barrios, J. A., Garcia Gil, S., Del Rosario Garcia, B., Nazco Casariego, G. J., Gutierrez Nicolas, F. | Efficacy and safety of cyclin dependent kinase inhibitors in metastatic breast cancer | 2021 | Population - mixed patient population or unclear whether population has failed CDK4/6i |
| Chainitikun, S., Long, J. P., Rodriguez-Bautista, R., Iwase, T., Tripathy, D., Fujii, T., Ueno, N. T. | The efficacy of first-line chemotherapy in endocrine-resistant hormone receptor-positive (HR+), human epidermal growth factor receptor 2-negative (HER2-) metastatic breast cancer | 2020 | Duplicate |
| Chainitikun, S., Long, J., Rodriguez-Bautista, R., Iwase, T., Tripathy, D., Fujii, T., Ueno, N. T. | The efficacy of first-line chemotherapy in endocrineresistant hormone receptorpositive (HR+), human epidermal growth factor receptor 2-negative (HER2-) metastatic breast cancer (MBC) | 2020 | Population - failed CDK4/6i but not in 1L |
| Chan, A., Lomma, C., Chih, H., Blackely, E., Woodward, N., Tsoi, D., Cheong, K., Chipman, M., Redfern, A. | Incorporation of eribulin in the systemic treatment of metastatic breast cancer patients in Australia | 2022 | Population - mixed patient population or unclear whether population has failed CDK4/6i |
| Chen, T., Zhang, Z., Gao, L., Scholz, C., Gualberto, A., Yu, L., Yu, K. | Using real-world data to evaluate the performance of endocrine therapies in ER+/Her2-metastatic breast cancer patients | 2022 | Population - failed CDK4/6i but not in 1L |
| Collins, J. M., Nordstrom, B. L., McLaurin, K. K., Dalvi, T. B., McCutcheon, S. C., Bennett, J. C., Murphy, B. R., Singhal, P. K., McCrea, C., Shinde, R., Briceno, J. M. | A Real-World Evidence Study of CDK4/6 Inhibitor Treatment Patterns and Outcomes in Metastatic Breast Cancer by Germline BRCA Mutation Status | 2021 | Population - mixed patient population or unclear whether population has failed CDK4/6i |
| Colon Lopez De Dicastillo, A., Gutierrez Perez, I., Villacanas Palomares, V., Uriarte Estefania, F., Lorenzo Martin, S., Santos Del Prado, R., Parra Alonso, E. | Real-world safety and tolerability of the recently commercialised palbociclib | 2019 | Population - mixed patient population or unclear whether population has failed CDK4/6i |
| Comis, S., Kandasamy, T., Wu, C., Kumar, S. P., Upadhyay, V. P., Chhikara, A. | Real-world evidence of CDK4/6 Inhibitor combination effects on assessing outcome with different sequence of treatment among adult patients with HR + and HER2 - metastatic breast cancer | 2020 | Population - mixed patient population or unclear whether population has failed CDK4/6i |
| Cook, M. M., Al Rabadi, L., Kaempf, A. J., Saraceni, M. M., Savin, M. A., Mitri, Z. I. | Everolimus Plus Exemestane Treatment in Patients with Metastatic Hormone Receptor-Positive Breast Cancer Previously Treated with CDK4/6 Inhibitor Therapy | 2021 | Population - failed CDK4/6i but not in 1L |
| Cook, M., Al Rabadi, L., Mitri, Z. I. | Everolimus and exemestane for the treatment of metastatic hormone receptor-positive breast cancer patients previously treated with CDK4/6 inhibitor based therapies | 2019 | Population - failed CDK4/6i but not in 1L |
| Coombes, C., Howell, S. J., Krebs, M. G., Lord, S., Kenny, L. M., Bahl, A., Clack, G., Ainscow, E., Dickinson, P. A., Fostea, R., Mansi, J., Palmieri, C., Bertelli, G., Jeselsohn, R., Mitri, Z., Gradishar, W. J., Sardesai, S., O'Shaughnessy, J., Ward, P., Chalasani, P., Lehnert, M., Ali, S., McIntosh, S. | Study of samuraciclib (CT7001), a first-in-class, oral, selective inhibitor of CDK7, in combination with fulvestrant in patients with advanced hormone receptor positive HER2 negative breast cancer (HR+BC) | 2022 | Study design |
| Crocetti, S., Pistelli, M., Pacenti, N., Tassone, L., Scortichini, L., Merloni, F., Agostinelli, V., De Filippis, C., Giuliani, L., Mammarella, A., Bastianelli, L., Cantini, L., Savini, A., Burattini, M., Lucarelli, A., Berardi, R. | The best therapeutic sequence for HR positive HER2 negative metastatic breast cancer after CDK4/6 inhibitors advent is still an open question? A single Institution experience | 2021 | Population - mixed patient population or unclear whether population has failed CDK4/6i |
| Dalal, A. A., Gagnon-Sanschagrin, P., Burne, R., Guerin, A., Gauthier, G., Small, T., Niravath, P. | Dosing Patterns and Economic Burden of Palbociclib Drug Wastage in HR+/HER2- Metastatic Breast Cancer | 2018 | Population - mixed patient population or unclear whether population has failed CDK4/6i |
| Dalal, A. A., Goldschmidt, D., Romdhani, H., Kelkar, S., Guerin, A., Wang, H., Caria, N., Sawhney, A., O'Shaughnessy, J. | Treatment patterns and sequences among pre-menopausal women with HR+/HER2-metastatic breast cancer: A chart review study | 2019 | Population - mixed patient population or unclear whether population has failed CDK4/6i |
| Dawood, S. S., Brzozowski, K. | Real-world PARPi treatment patterns and outcomes among patients with metastatic breast cancer | 2022 | Population - failed CDK4/6i but not in 1L |
| Dawood, S., Brzozowski, K. | Use of PARPi among patients with advanced breast cancer | 2021 | Population - failed CDK4/6i but not in 1L |
| De Luna Aguilar, A., Moreno Anton, F., Benitez Fuentes, J. D., Ortega Anselmi, J., Olalla Inoa, J., Flores Navarro, P., Garcia Saenz, J. A. | Abemaciclib in HR+/Her2- metastatic breast cancer patients after previous progression on palbociclib or ribociclib: Clinical experience in a tertiary hospital in Madrid, Spain | 2022 | Population - failed CDK4/6i but not in 1L |
| Decker, T., Seifert, R., Bichler, M., Birtel, A., Fischer, G., Nonnenbroich, C., Dechow, T. | Elective Discontinuation of CDK4/6 Inhibitors in Patients with Metastatic Hormone Receptor-Positive, Her-2-Negative Breast Cancer: A Retrospective Single-Center Experience | 2021 | Population - failed CDK4/6i but not in 1L |
| Demir, A., Mandel, N. M., Paydas, S., Demir, G., Er, O., Turhal, N. S., Bavbek, S., Eralp, Y., Saip, P. M., Guler, E. N., Aydiner, A., Uluc, B. O., Kilickap, S., Uskent, N., Karadurmus, N., Kaplan, M. A., Yanmaz, M. T., Demir, H., Alan, O., Korkmaz, T., Olgun, P., Uysal, O. S., Altundag, K., Gunduz, S., Gunaldi, M., Sari, M., Beypinar, I., Basaran, G. | Efficacy of palbociclib and endocrine treatment in heavily pretreated hormone receptor-positive/HER2-negative advanced breast cancer: Retrospective multicenter trial | 2020 | Population - mixed patient population or unclear whether population has failed CDK4/6i |
| DeSouza, K., Yeo, D., Diossy, M., Umar, S., Gore, E., Trivedi, S., Anand, A., Madhusudan, S., Khan, S. | Real-world outcomes from the systemic use of CDK 4/6 inhibitors (CDKIs) in the management of ER positive (+) HER2 negative (-) metastatic breast cancer (mBC) | 2021 | Population - mixed patient population or unclear whether population has failed CDK4/6i |
| Dhakal, A., Antony Thomas, R., Levine, E. G., Brufsky, A., Takabe, K., Hanna, M. G., Attwood, K., Miller, A., Khoury, T., Early, A. P., Soniwala, S., O'Connor, T., Opyrchal, M. | Outcome of Everolimus-Based Therapy in Hormone-Receptor-Positive Metastatic Breast Cancer Patients After Progression on Palbociclib | 2020 | Population - failed CDK4/6i but not in 1L |
| Dos Anjos, C. H., Razavi, P., Herbert, J., Colon, J., Gill, K., Modi, S., Bromberg, J., Dang, C. T., Liu, D., Norton, L., Chandarlapaty, S., Robson, M. E., Jhaveri, K. L. | A large retrospective analysis of CDK 4/6 inhibitor retreatment in ER+ metastatic breast cancer (MBC) | 2019 | Population - failed CDK4/6i but not in 1L |
| du Rusquec, P., Palpacuer, C., Campion, L., Patsouris, A., Augereau, P., Gourmelon, C., Robert, M., Dumas, L., Caroline, F., Campone, M., Frenel, J. S. | Efficacy of palbociclib plus fulvestrant after everolimus in hormone receptor-positive metastatic breast cancer | 2018 | Population - failed CDK4/6i but not in 1L |
| Fabi, A., Giannarelli, D., Botticelli, A., Scagnoli, S., Pellegrino, A., Fabbri, A., Corsi, D., Magri, V., Pizzuti, L., Paris, I., Bruni, V., Pace, R., Lanzetta, G., Stani, S., Moscetti, L., Marchetti, P., Piesco, G., Cognetti, F., Rossi, V. | SEQUERPLUS: A multicenter real practice observational study investigating the endocrine-based (E) therapies sequential approach in hormonal receptor positive (HR+) HER2 negative (-) metastatic breast cancer (MBC) | 2019 | Population - mixed patient population or unclear whether population has failed CDK4/6i |
| Falcon, A., Bofill, J. S., Montano, A., Benavent, M., Gonzalez, R., Borrego, M. R. | CDK4/6 inhibitors beyond second line, is it worthy? A real-world data experience | 2020 | Population - mixed patient population or unclear whether population has failed CDK4/6i |
| Fernandez-Cuerva, C., del Rio Valencia, J. C., Bermejo, R. T. | Effectiveness and Safety of Palbociclib plus Endocrine Therapy in Hormone Receptor- Positive, HER2-Negative Metastatic Breast Cancer: Real-World Results | 2022 | Population - mixed patient population or unclear whether population has failed CDK4/6i |
| File, D. M., Pascual, T., Deal, A. M., Wheless, A., Perou, C. M., Claire Dees, E., Carey, L. A. | Clinical subtype, treatment response, and survival in De Novo and recurrent metastatic breast cancer | 2022 | Population - mixed patient population or unclear whether population has failed CDK4/6i |
| Fountzilas, E., Koliou, G. A., Rapti, V., Nikolakopoulos, A., Christopoulou, A., Moirogiorgou, E., Binas, I., Aravantinos, G., Kostadima, L., Nikolaidi, A., Karteri, S., Zagouri, F., Saridaki, Z., Molfeta, A., Oikonomopoulou, P., Res, E., Tryfonopoulos, D., Koumakis, G., Fountzilas, G., Razis, E. | Clinical outcome and toxicity data in patients with advanced breast cancer treated with cyclin-dependent kinase 4/6 (CDK4/6) inhibitors combined with endocrine therapy in a real-world clinical setting | 2019 | Population - mixed patient population or unclear whether population has failed CDK4/6i |
| Fountzilas, E., Koliou, G. A., Vozikis, A., Rapti, V., Nikolakopoulos, A., Boutis, A., Christopoulou, A., Kontogiorgos, I., Karageorgopoulou, S., Lalla, E., Tryfonopoulos, D., Boukovinas, I., Rapti, C., Nikolaidi, A., Karteri, S., Moirogiorgou, E., Binas, I., Mauri, D., Aravantinos, G., Zagouri, F., Saridaki, Z., Psyrri, A., Bafaloukos, D., Koumarianou, A., Res, E., Linardou, H., Mountzios, G., Razis, E., Fountzilas, G., Koumakis, G. | Real-world clinical outcome and toxicity data and economic aspects in patients with advanced breast cancer treated with cyclin-dependent kinase 4/6 (CDK4/6) inhibitors combined with endocrine therapy: The experience of the Hellenic Cooperative Oncology Group | 2020 | Population - mixed patient population or unclear whether population has failed CDK4/6i |
| Fuentes-Antras, J., de Luna, A., Lopez de Sa, A., Ocana, A., Garcia-Saenz, J. A., Moreno, F. | Safety and efficacy of cyclin-dependent kinase inhibitor rechallenge following ribociclib-induced limiting hypertransaminasemia | 2020 | Population - failed CDK4/6i but not in 1L |
| Fushimi, A., Tabei, I., Fuke, A., Okamoto, T., Takeyama, H. | High-Dose Toremifene as a Promising Candidate Therapy for Hormone Receptor-Positive Metastatic Breast Cancer with Secondary Resistance to Aromatase Inhibitors | 2020 | Population - mixed patient population or unclear whether population has failed CDK4/6i |
| Garcia-Trevijano Cabetas, M., Lucena Martinez, P., Jimenez Nacher, I., Diaz Almiron, M., Zamora Aunon, P., Herrero Ambrosio, A. | Real-world experience of palbociclib and ribociclib: novel oral therapy in metastatic breast cancer | 2021 | Population - mixed patient population or unclear whether population has failed CDK4/6i |
| Garcia-Trevijano Cabetas, M., Lucena, P., Jimenez-Nacher, I., Villamanan, E., Sobrino, C., Bilbao, C., Zamora, P., Herrero, A. | Real world effectiveness of palbociclib and ribociclib in women with metastatic breast cancer | 2021 | Population - mixed patient population or unclear whether population has failed CDK4/6i |
| Gelmon, K. A., Fasching, P. A., Couch, F. J., Balmana, J., Delaloge, S., Labidi-Galy, I., Bennett, J., McCutcheon, S., Walker, G., O'Shaughnessy, J., Timcheva, C., Tomova, A., Eisen, A., Lemieux, J., Bazan, F., Bourgeois, H., Chakiba, C., Chehimi, M., Dalenc, F., De La Motte Rouge, T., Frenel, J. S., Goncalves, A., Hardy-Bessard, A. C., Lamy, R., Levy, C., Lortholary, A., Mailliez, A., Medioni, J., Patsouris, A., Spaeth, D., Teixeira, L., Tennevet, I., Villanueva, C., You, B., Ettl, J., Gerber, B., Hoffmann, O., Park-Simon, T. W., Reinisch, M., Tio, J., Wimberger, P., Boer, K., Ballestrero, A., Bianchini, G., Biganzoli, L., Bordonaro, R., Cognetti, F., De Laurentiis, M., De Placido, S., Guarneri, V., Montemurro, F., Naso, G., Santoro, A., Zamagni, C., Kim, S. J., Nakamura, S., Chae, Y. S., Cho, E. K., Hyun, K. J., Im, S. A., Lee, K. S., Park, Y. H., Sohn, J. H., Byrski, T., Huzarski, T., Kukielka-Budny, B., Nowecki, Z., Szoszkiewicz, R., Tarnawski, R., Dvornichenko, V., Moiseenko, F., Mukhametshina, G., Poddubskaya, E., Popova, E., Tarasova, A., Vats, A., Adamo, B., Conejero, R. A., Torres, A. A., Gelpi, J. B., Fernandez, N. D., Gonzalez, A. F., Garcia, J., Lorenzo-Lorenzo, I., Anton, F. M., Santisteban, M., Stradella, A., Huang, C. S., Aksoy, S., Arslan, C., Artac, M., Aydiner, A., Ozyilkan, O., Sezer, E., Armstrong, A., Barrett, S., Borley, A., Kemp, Z., Michie, C., Mukesh, M., Perren, T., Swampillai, A., Young, T. | Clinical effectiveness of olaparib monotherapy in germline BRCA-mutated, HER2-negative metastatic breast cancer in a real-world setting: phase IIIb LUCY interim analysis | 2021 | Population - failed CDK4/6i but not in 1L |
| Gharib, K. E., Macaron, W., Kattan, J., Salloum, M. A., Farhat, F., Smith, M., Karak, F. E. | Palbociclib and letrozole in hormone-receptor positive advanced breast cancer: Predictive response and prognostic factors | 2022 | Population - mixed patient population or unclear whether population has failed CDK4/6i |
| Goldschmidt, D., Dalal, A. A., Romdhani, H., Kelkar, S., Guerin, A., Gauthier, G., Wu, E. Q., Niravath, P., Small, T. | Current Treatment Patterns Among Postmenopausal Women with HR+/HER2- Metastatic Breast Cancer in US Community Oncology Practices: An Observational Study | 2018 | Population - mixed patient population or unclear whether population has failed CDK4/6i |
| Ha, M. J., Singareeka Raghavendra, A., Kettner, N. M., Qiao, W., Damodaran, S., Layman, R. M., Hunt, K. K., Shen, Y., Tripathy, D., Keyomarsi, K. | Palbociclib plus endocrine therapy significantly enhances overall survival of HR+/HER2- metastatic breast cancer patients compared to endocrine therapy alone in the second-line setting: A large institutional study | 2022 | Population - mixed patient population or unclear whether population has failed CDK4/6i |
| Harbeck, N., Bartlett, M., Spurden, D., Hooper, B., Zhan, L., Rosta, E., Cameron, C., Mitra, D., Zhou, A. | PCN28 CYCLIN-DEPENDENT KINASE 4/6 INHIBITORS (CDK4/6I) IN HORMONE RECEPTOR-POSITIVE/HUMAN EPIDERMAL GROWTH FACTOR RECEPTOR 2-NEGATIVE (HR+/HER2-) ADVANCED/METASTATIC BREAST CANCER (A/MBC): A SYSTEMATIC LITERATURE REVIEW OF REAL-WORLD EVIDENCE (RWE) STUDIES | 2020 | On topic SLR/MA/NMA |
| Hayama, S., Nakamura, R., Miyaki, T., Itami, M., Yamamoto, N. | Treatment Strategy for Patients with HR-Positive HER2-Negative Metastatic Breast Cancer That Progressed on CDK4/6 Inhibitors | 2022 | Population - failed CDK4/6i but not in 1L |
| Herrscher, H., Velten, M., Leblanc, J., Kalish-Weindling, M., Fischbach, C., Exinger, D., Pivot, X., Petit, T. | Fulvestrant and palbociclib combination in heavily pretreated hormone receptor-positive, HER2-negative metastatic breast cancer patients | 2020 | Population - mixed patient population or unclear whether population has failed CDK4/6i |
| Hester, A., Koenig, A., Dobler, F., Degenhardt, T., Heidegger-Steger, H., Kurt, A. G., Kahlert, S., Mahner, S., Harbeck, N., Wuerstlein, R. | Palbociclib in daily clinical use: Real world experience of the breast center at the University Hospital Munich | 2019 | Population - mixed patient population or unclear whether population has failed CDK4/6i |
| Hoste, G., Punie, K., Wildiers, H., Beuselinck, B., Lefever, I., Van Nieuwenhuysen, E., Han, S. N., Berteloot, P., Concin, N., Salihi, R., Vergote, I., Neven, P. | Palbociclib in highly pretreated metastatic ER-positive HER2-negative breast cancer | 2018 | Population - mixed patient population or unclear whether population has failed CDK4/6i |
| Hoste, G., Slembrouck, L., Jongen, L., Punie, K., Matton, T., Vander Borght, S., Vanden Bempt, I., Menten, J., Wildiers, H., Floris, G., Arteaga, C., Neven, P. | Unexpected Benefit from Alpelisib and Fulvestrant in a Woman with Highly Pre-treated ER-Positive, HER2-Negative PIK3CA Mutant Metastatic Breast Cancer | 2018 | Population - failed CDK4/6i but not in 1L |
| Howell, S. J., Krebs, M. G., Lord, S., Kenny, L., Bahl, A., Clack, G., Ainscow, E., Arkenau, H. T., Mansi, J. L., Palmieri, C., Richards, P., Jeselsohn, R., Mitri, Z., Gradishar, W. J., Sardesai, S., O'Shaughnessy, J., Lehnert, M., Ali, S., McIntosh, S., Coombes, R. C. | Study of samuraciclib (CT7001), a first-in-class, oral, selective inhibitor of CDK7, in combination with fulvestrant in patients with advanced hormone receptor positive HER2 negative breast cancer (HR+BC) | 2021 | Duplicate |
| Isca, C., Piacentini, F., Bocconi, A., Toss, A., Barbieri, E., Cortesi, L., Moscetti, L., Cascinu, S., Omarini, C. | Safety and efficacy of CDK4/6 inhibitors in patients with advanced breast cancer: A real word experience | 2019 | Population - mixed patient population or unclear whether population has failed CDK4/6i |
| Jeong, H., Jeong, J. H., Kim, J. E., Ahn, J. H., Jung, K. H., Kim, S. B. | Comparison of the Effectiveness and Clinical Outcome of Everolimus Followed by CDK4/6 Inhibitors with the Opposite Treatment Sequence in Hormone Receptor-Positive, HER2-Negative Metastatic Breast Cancer | 2022 | Population - failed CDK4/6i but not in 1L |
| Jeong, J. H., Jeong, H., Kim, J. E., Ahn, J. H., Jung, K. H., Kim, S. B. | Clinical efficacy of everolimus and CDK4/6 inhibitors in hormone receptor-positive, HER2-negative metastatic breast cancer by treatment sequence | 2021 | Population - failed CDK4/6i but not in 1L |
| Karuturi, M., Blum, J. L., Anderson, D., Kurian, S., Wilks, S. T., Wang, G., Gauthier, E., Zhang, Z., Wang, Y., Tripathy, D., Rocque, G. | Palbociclib treatment in pre/perimenopausal women with advanced/metastatic breast cancer (ABC/mBC): Real-world patient characteristics, treatment patterns, and outcomes data from POLARIS | 2022 | Population - mixed patient population or unclear whether population has failed CDK4/6i |
| Kawai, M., Takada, M., Nakayama, T., Masuda, N., Shiheido, H., Cai, Z., Huang, Y. J., Kawaguchi, T., Tanizawa, Y. | Patient characteristics, treatment patterns, and outcomes of hormone receptor-positive, human epidermal growth factor receptor 2-negative advanced breast cancer patients prescribed cyclin-dependent kinase 4 and 6 inhibitors: large-scale data analysis using a Japanese claims database | 2022 | Population - failed CDK4/6i but not in 1L |
| Kish, J. K., Ward, M. A., Garofalo, D., Ahmed, H. V., McRoy, L., Laney, J., Zanotti, G., Braverman, J., Yu, H., Feinberg, B. A. | Real-world evidence analysis of palbociclib prescribing patterns for patients with advanced/metastatic breast cancer treated in community oncology practice in the USA one year post approval | 2018 | Population - mixed patient population or unclear whether population has failed CDK4/6i |
| Kitano, S., Honda, A., Itoi, N., Lee, T. | Everolimus for Treating Hormone Receptor-positive Metastatic Breast Cancer Previously Treated With Cyclin-dependent Kinase 4/6 Inhibitors | 2022 | Population - failed CDK4/6i but not in 1L |
| Kolyadina, I. V., Bolotina, L., Zhukova, L., Vladimirova, L. Y., Sultanbaev, A., Karabina, E., Ganshina, I., Ovchinnikova, E., Antonova, G., Volkonsky, M., Kolokolov, J., Zueva, E., Akopyan, I., Fadeeva, N., Evstigneeva, I., Orlova, S., Vasilevskaya, A., Shalaeva, O., Shirokova, O. | The effectiveness and safety of eribulin therapy in HR-positive HER2-negative metastatic breast cancer post-CDK4/6 inhibitor therapy in Russian clinical practice | 2021 | Population - failed CDK4/6i but not in 1L |
| Kovac, A. N. J. A., Matos, E., Kuhar, C. G., Caks, M., Ovcaricek, T., Mencinger, M., Humar, M., Borstnar, S. | Efficacy and safety of selective cyclin-dependent kinases 4/6 inhibitors in hormone-receptor-positive, HER2-negative advanced breast cancer - results from a real-world setting | 2020 | Population - mixed patient population or unclear whether population has failed CDK4/6i |
| Kubeczko, M., Badora-Rybicka, A., Polakiewicz-Gilowska, A., Lesniak, A., Swiderska, K., Mianowska-Malec, M., Grandys, B., Lanoszka, B., Stankiewicz, M., Gawkowska, M., Nowicka, E., Wozniak, G., Gabrys, D., Tarnawski, R., Miszczyk, L., Jarzqb, M. | Adverse events in breast cancerpatients treated with concurrent or sequential radiationtherapy and CDK 4/6 inhibitors in metastatic setting | 2021 | Population - mixed patient population or unclear whether population has failed CDK4/6i |
| Kurbacher, C. M., Fischer, L. A., Heinrich, G., Herz, S., Schott, A., Kurbacher, A. T., Kurbacher, J. A., Rudlowski, C., Warm, M. R. | Application of CDK4/6 inhibitors in premenopausal women with hormone receptor-positive, HER2-negative metastatic breast cancer: A real-world experience | 2020 | Population - mixed patient population or unclear whether population has failed CDK4/6i |
| Kurbacher, C. M., Fischer, L. A., Heinrich, G., Warm, M., Schott, A., Tabea Kurbacher, A., Kurbacher, J. A., Rudlowski, C. | TREATMENT OF PREMENOPAUSAL PATIENTS WITH HORMONE RECEPTOR-POSITIVE, HER2-NEGATIVE METASTATIC BREAST CANCER WITH AN CDK4/6 INHIBITOR COMBINED WITH ENDOCRINE AGENTS: A REAL-WORLD EXPERIENCE | 2019 | Population - mixed patient population or unclear whether population has failed CDK4/6i |
| Laguna, J. C., Braso-Maristany, F., Pascual, T., Rodriguez Hernandez, A., Chic, N., Schettini, F., Sanfeliu Torres, E., Gonzalez-Farre, B., Martinez, D., Galvan, P., Diez-Guardia, V., Adamo, B., Vidal, M., Guillen Sacoto, M. C., Moreno, R., Prat, A., Munoz, M., Martinez-Saez, O. | Subsequent therapies after progressing to CDK4/6 inhibition (CDK4/6i) in hormone receptor positive/HER2 negative (HR+/HER2-) advanced breast cancer (ABC) | 2021 | Population - failed CDK4/6i but not in 1L |
| Lama Tamang, T. G., Kyung, D., Eisenbud, L., Tang, T., Parajuli, R., Mehta, R. S. | Use of alpelisib in the treatment of hormone receptor positive metastatic breast cancer: An institutional experience | 2020 | Population - failed CDK4/6i but not in 1L |
| Lana, J. B., Josipa, J. Z. | CASE REPORT: THE PATIENT INITIALLY DIAGNOSED WITH HORMONE RECEPTOR POSITIVE, HER 2 NEGATIVE METASTATIC BREAST CANCER (HR+/HER2- MBC) WITH AQUIRED RESISTANCE TO ENDOCRINE THERAPY | 2022 | Population - failed CDK4/6i but not in 1L |
| Landry, C. A., Ru, M., Jaffer, S., Dimitrova, M., Tiersten, A. | The significance of androgen receptor co-expression in ER+ metastatic breast cancer patients treated with palbociclib | 2019 | Population - mixed patient population or unclear whether population has failed CDK4/6i |
| Lewis, K., Kurosky, S., Last, M., Mitra, D., Lambert, A., Mahtani, R. | Treatment sequencing in HR+/HER2- locally advanced or metastatic breast cancer: A real-world retrospective study in France, Germany, Italy and Spain | 2020 | Outcome (e.g., only reports patient-reported outcomes/utilities) |
| Li, J., Zhang, X., Yang, C., Lv, Y., Yang, H., Kong, X., Han, M., Wang, Z., Ma, J., Han, J., Liu, Y. | Real-world effectiveness and sensitivity of palbociclib plus endocrine therapy in HR+/HER2-patients with metastatic breast cancer | 2021 | Population - mixed patient population or unclear whether population has failed CDK4/6i |
| Limardi, S., Summaria, E., Spadafora, F., Miceli, C., Scura, R., Conforti, M. R., Zagarese, M. | Breast Cancer Hormone-Positive/HER2 negative: Real-life data of everolimus/exemestan and palbociclib/fulvestrant associations and comparison with BOLERO-2 e PALOMA-3 experimental studies | 2021 | Non-English |
| Little, J., Burcombe, R., Parsons, E., Ryan, C. | Eribulin Use and Palliative Care Referral Rates in Metastatic Breast Cancer: kent Oncology Centre Experience | 2020 | Population - mixed patient population or unclear whether population has failed CDK4/6i |
| Liu, C., Li, T., Tao, Z., Cao, J., Wang, L., Zhang, J., Wang, B., Hu, X. | Clinical outcomes of 130 patients with hormone receptor-positive and human epidermal growth factor receptor 2-negative metastatic breast cancer treated with palbociclib plus endocrine therapy and subsequent therapy: A real-world single-center retrospective study in China | 2020 | Population - failed CDK4/6i but not in 1L |
| Loirat, D., De Labarre, M. D., Essner, C., Hrab, I., Thery, J. C., Jouannaud, C., Villanueva, C., Vuagnat, P., Soibinet-Oudot, P., Creisson, A., Mailliez, A., Mouysset, J. L., Salabert, L., Dohollou, N., Fumet, J. D., De La Motte Rouge, T., Vauthier, J. M., Decrop, M., Pujol, P. | Phase IV study evaluating effectiveness and safety of talazoparib in patients with locally advanced or metastatic HER2 negative breast cancer and a BRCA1 or BRCA2 mutation (ViTAL) | 2022 | Population - mixed patient population or unclear whether population has failed CDK4/6i |
| Luca Battisti, N. M., Morrison, L., Nash, T., Senthivel, N., Kestenbaum, S., Begum, P., Obeid, M., Hayhurst, W., Yang, D., Gafoor, S., Brown, C., Rehman, F., Kenny, L., Hatcher, O., Susan, S., Williams, J., Brown, A., Rozati, H., Alexandros, A., Sawyer, E., Gousis, C., Karapanagiotou, E., Rigg, A., Rapti, K., Roylance, R., Beresford, M., Gee, A. L., Konstantis, A., King, J., Nathan, M., Spurrell, E., Pearce, M., Bradwell, D., Denton, A., Swain, K., McGrath, S., Allen, M., Ring, A., Johnston, S., Raja, F. | Abemaciclib and endocrine therapy for hormone receptor-positive, HER2-negative advanced breast cancer: A real-world UK multicentre experience | 2022 | Population - mixed patient population or unclear whether population has failed CDK4/6i |
| Luftner, D., Brucker, C., Decker, T., Fasching, P., Gohler, T., Jackisch, C., Janssen, J., Kohler, A., Ludtke-Heckenkamp, K., Van MacKelenbergh, M., Marme, F., Nusch, A., Rautenberg, B., Reimer, T., Schmidt, M., Weide, R., Wimberger, P., Nabieva, N., Roos, C., Wockel, A. | Real-world efficacy of ribociclib + aromatase inhibitor/fulvestrant, or endocrine monotherapy, or chemotherapy as first-line treatment in women with hormone receptor-positive (HR+), human epidermal growth factor receptor-2-negative (HER2-) locally advanced or metastatic breast cancer: fourth interim analysis from the RIBANNA study | 2022 | Population - mixed patient population or unclear whether population has failed CDK4/6i |
| Lupichuk, S. M., Recaldin, B., Nixon, N. A., Mututino, A., Joy, A. A. | Real-world experience using exemestane and everolimus in patients with hormone receptor positive/HER2 negative breast cancer with and without prior CDK4/6 inhibitor exposure | 2019 | Population - failed CDK4/6i but not in 1L |
| Lux, M. P., Runkel, E. D., Glastetter, E., Vannier, C., Buncke, J., Frank, M., Bartsch, R., Thill, M., Wockel, A. | PERFORM: a non-interventional study assessing the patients' treatment starting with 1L palbociclib in HR+/HER2- ABC | 2022 | Incomplete/Insufficient/Partial data |
| Manso, L., Bermejo, B., Delgado, I., Aguirre, E., Oltra, A., Gonzalez, M., Malon, D., Ales, J. E., Rodriguez, C., Moreno, F. | PALBOCOMP: Retrospective observational analysis of palbociclib treatment in patients with advanced breast cancer within a compassionate use program in Spain | 2019 | Population - mixed patient population or unclear whether population has failed CDK4/6i |
| Manso, L., Hernando, C., Galan, M., Oliveira, M., Cabrera, M. A., Bratos, R., Rodriguez, C. A., Ruiz-Borrego, M., Blanch, S., Llombart-Cussac, A., Delgado-Mingorance, J. I., Alvarez-Busto, I., Gallegos, I., Gonzalez-Cortijo, L., Morales, S., Aguirre, E., Hernando, B. A., Ballesteros, A., Ales-Martinez, J. E., Reboredo, C., Oltra, A., Gonzalez-Cao, M., Santisteban, M., Malon, D., Echeverria, I., Garcia-Garre, E., Vega, E., Servitja, S., Andres, R., Robles, C. E., Lopez, R., Galve, E., Echarri, M. J., Legeren, M., Moreno, F. | Palbociclib combined with endocrine therapy in heavily pretreated HR+/HER2- advanced breast cancer patients: Results from the compassionate use program in Spain (PALBOCOMP) | 2020 | Population - mixed patient population or unclear whether population has failed CDK4/6i |
| Mar Munoz Sanchez, M. D., Carmen Soriano Rodriguez, M. D., Molina Garrido, M. J., Lopez-Gonzalez, A., Garcia-Palomo, A., Lopez-Gonzalez, L., Plata Fernandez, M. Y., Caro, N. L., Rovira, P. S. | Experience with eribulin in HR+/HER2- metastatic breast cancer, including a male | 2018 | Population - mixed patient population or unclear whether population has failed CDK4/6i |
| Mariotti, V., Khong, H. T., Soliman, H. H., Costa, R. L., Fisher, S., Boulware, D., Han, H. S. | Efficacy of abemaciclib (abema) after palbociclib (palbo) in patients (pts) with metastatic breast cancer (MBC) | 2019 | Population - failed CDK4/6i but not in 1L |
| Marra, A., Gazzo, A., Gupta, A., Selenica, P., Da Silva, E. M., Pareja, F., Pei, X., Zhu, Y., Razavi, P., Safonov, A., Ferraro, E., Harris, R., Riaz, N., Reis-Filho, J. S., Chandarlapaty, S. | Mutational signature analysis reveals patterns of genomic instability linked to resistance to endocrine therapy (ET) +/- CDK 4/6 inhibition (CDK4/6i) in estrogen receptor-positive/HER2-negative (ER+/HER2-) metastatic breast cancer (MBC) | 2022 | Population - mixed patient population or unclear whether population has failed CDK4/6i |
| Maurer, C., Ferreira, A. R., Martel, S., Lambertini, M., Ponde, N., Aftimos, P., de Azambuja, E., Piccart, M. | Endocrine therapy and palbociclib within a compassionate use program in heavily pretreated hormone receptor-positive, HER2-negative metastatic breast cancer | 2018 | Population - mixed patient population or unclear whether population has failed CDK4/6i |
| Mavratzas, A., Marme, F. | Treatment of Luminal Metastatic Breast Cancer beyond CDK4/6 Inhibition: is There a Standard of Care in Clinical Practice? | 2021 | Study design |
| McLaurin, K., Dalvi, T., Collins, J. M., Nordstrom, B. L., McCutcheon, S., Bennett, J. C., Murphy, B. R., Singhal, P. K., Briceno, J. M. | A real-world evidence study of CDK4/6 inhibitor treatment patterns and outcomes in metastatic breast cancer by gBRCA mutation status | 2019 | Population - mixed patient population or unclear whether population has failed CDK4/6i |
| Meattini, I., Desideri, I., Scotti, V., Simontacchi, G., Livi, L. | Ribociclib plus letrozole and concomitant palliative radiotherapy for metastatic breast cancer | 2018 | Population - mixed patient population or unclear whether population has failed CDK4/6i |
| Meegdes, M., Geurts, S. M. E., Erdkamp, F. L. G., Dercksen, M. W., Vriens, B. E. P. J., Aaldering, K. N. A., Pepels, M. J. A. E., van de Winkel, L. M. H., Teeuwen, N. J. A., de Boer, M., Tjan-Heijnen, V. C. G. | The implementation of CDK 4/6 inhibitors and its impact on treatment choices in HR+/HER2- advanced breast cancer patients: A study of the Dutch SONABRE Registry | 2022 | Population - mixed patient population or unclear whether population has failed CDK4/6i |
| Miller, J., Armgardt, E., Svoboda, A. | The efficacy and safety of alpelisib in breast cancer: A real-world analysis | 2022 | Population - failed CDK4/6i but not in 1L |
| Miyahara, Kana, Narui, Kazutaka, Uemura, Yukari, Yamada, Akimitsu, Araki, Kazuhiro, Fujisawa, Fumie, Nakayama, Takahiro, Ishikawa, Takashi, Taira, Naruto, Kikawa, Yuichiro, Aihara, Tomohiko, Mukai, Hirofumi | Prospective Cohort Study of Combination Therapy With Abemaciclib and Hormonal Therapy for Chemotherapy-Treated Patients With Hormone Receptor-Positive Metastatic Breast Cancer | 2022 | Incomplete/Insufficient/Partial data |
| Mo, H., Renna, C. E., Moore, H. C. F., Abraham, J., Kruse, M. L., Montero, A. J., LeGrand, S. B., Wang, L., Budd, G. T. | Real-World Outcomes of Everolimus and Exemestane for the Treatment of Metastatic Hormone Receptor-Positive Breast Cancer in Patients Previously Treated With CDK4/6 Inhibitors | 2022 | Population - failed CDK4/6i but not in 1L |
| Mo, H., Renna, C. E., Moore, H. C., Abraham, J., Kruse, M. L., Montero, A. J., LeGrand, S. B., Budd, G. T. | Efficacy of everolimus andexemestane for the treatment of metastatic hormonereceptor-positive breast cancer in patients previouslytreated with CDK4/6 inhibitors | 2021 | Duplicate |
| Moreira, I., Ferreira, M., Afonso, A., Ferreira, A., Rodrigues, A., Vieira, C., Oliveira, C., SavvaBordalo, J., Dias, J., Cassiano, M., Abreu, M., Alves, S., Sousa, S. | Everolimus and exemestane in hormone receptor-positive advanced breast cancer: A comprehensive cancer center's experience | 2021 | Population - mixed patient population or unclear whether population has failed CDK4/6i |
| Mougalian, S. S., Zhang, J., Kish, J. K., Zettler, M. E., Feinberg, B. A. | Real-world treatment patterns and clinical effectiveness of eribulin in HR+/HER2- metastatic breast cancer patients in the United States | 2021 | Population - failed CDK4/6i but not in 1L |
| Murali, B., Durbin, L., Vijaykumar, S., Yang, L., Li, S., Zhao, L., Hawthorne, S., Kanas, G., Davis, C., Clark, O. | Treatment of HR+/HER2- breast cancer in urban mainland China: results from the CancerMPact Survey 2019 | 2022 | Population - mixed patient population or unclear whether population has failed CDK4/6i |
| Mycock, K., Hanson, K. A., Taylor-Stokes, G., Milligan, G., Atkinson, C., Mitra, D., Preciado, S., Law, E. H. | Real-World Treatment Patterns and Clinical Outcomes Associated With Palbociclib Combination Therapy: A Multinational, Pooled Analysis From the Ibrance Real World Insights Study | 2022 | Population - mixed patient population or unclear whether population has failed CDK4/6i |
| Mycock, K., Hanson, K., Taylor-Stokes, G., Milligan, G., Atkinson, C., Mitra, D., Preciado, S. M., Law, E. | POSB400 Real World Treatment Patterns and Clinical Outcomes Associated with Palbociclib Combination Therapy across Europe, North and South America, and ASIA: A Pooled Analysis from the IRIS Study | 2022 | Population - mixed patient population or unclear whether population has failed CDK4/6i |
| Nakano, S., Imawari, Y., Mibu, A., Kato, S., Yamaguchi, S., Otsuka, M., Sano, M. | Molecular Targeted Therapy for Hormone Receptor-Positive, Human Epidermal Growth Factor 2-Negative Metastatic Breast Cancer in Clinical Practice | 2022 | Population - failed CDK4/6i but not in 1L |
| Nasr, L., Ghoche, A., Diab, S., Nasr, F. | Real-world survival data of palbociclib in advanced and metastatic breast cancer: A multicenter experience in Lebanon | 2020 | Population - mixed patient population or unclear whether population has failed CDK4/6i |
| Navarro-Yepes, J., Kettner, N. M., Bui, T., Raghavendra, A. S., Rao, X., Wang, J., Sahin, A., Damodaran, S., Tripathy, D., Hunt, K. K., Keyomarsi, K. | Mechanisms of acquired resistance to palbociclib reveals pathways of response to abemaciclib | 2022 | Population - failed CDK4/6i but not in 1L |
| Nawaz, A., Zekri, J., Rasool, H. J. | Initial real life experience with ribociclib in breast cancer: Outcome with focus on tolerance and cardiac toxicity | 2021 | Population - mixed patient population or unclear whether population has failed CDK4/6i |
| Novick, Diego, Lee, Sae Young, Koo, Dong Hyun, Szende, Agota, Colman, Sam | Real world evidence study on treatment patterns and health resource utilization in patients with HR+/HER2- locally advanced or metastatic breast cancer in Korea | 2022 | Population - all other reasons |
| Odan, N., Kikawa, Y., Matsumoto, H., Minohata, J., Suwa, H., Hashimoto, T., Okuno, T., Miyashita, M., Saito, M., Yamagami, K., Takao, S. | Real-World Outcomes of Treating Advanced Breast Cancer Patients With Palbociclib: A Multicenter Retrospective Cohort Study in Japan-The KBCOG-14 Study | 2020 | Population - mixed patient population or unclear whether population has failed CDK4/6i |
| Odan, N., Kikawa, Y., Matsumoto, H., Minohata, J., Suwa, H., Hashimoto, T., Okuno, T., Miyashita, M., Saito, M., Yamagami, K., Takao, S. | REAL-WORLD OUTCOMES OF PATIENTS WITH ADVANCED BREAST CANCER TREATED WITH PALBOCICLIB: A MULTICENTER RETROSPECTIVE COHORT STUDY IN JAPAN | 2019 | Population - mixed patient population or unclear whether population has failed CDK4/6i |
| Oliveira, C., Redondo, P., Fonseca, M. J., Oliveira Gomes, J., Sousa, S., Pereira, D. | PCN27 Treatment Outcomes and Healthcare Resources Utilization of Palbociclib in Advanced Breast Cancer | 2020 | Population - mixed patient population or unclear whether population has failed CDK4/6i |
| Orlandi, A., Iattoni, E., Pizzuti, L., Fabbri, A., Botticelli, A., Di Dio, C., Palazzo, A., Garufi, G., Indellicati, G., Alesini, D., Carbognin, L., Paris, I., Vaccaro, A., Magri, V., Naso, G., Cassano, A., Vici, P., Giannarelli, D., Marchetti, P., Bria, E., Tortora, G. | Palbociclib-fulvestrant (PALBO-FUL) and everolimus-exemestane (EVE-EXE) for second line hormonal treatment (HT) of metastatic breast cancer (MBC) with lobular histology: A propensity score matched analysis | 2019 | Duplicate |
| Orlandi, A., Iattoni, E., Pizzuti, L., Fabbri, A., Botticelli, A., Di Dio, C., Palazzo, A., Garufi, G., Indellicati, G., Alesini, D., Carbognin, L., Paris, I., Vaccaro, A., Moscetti, L., Cassano, A., Vici, P., Magri, V., Naso, G., Giannarelli, D., Marchetti, P., Bria, E., Tortora, G. | Palbociclib-fulvestrant (PALBO-FUL) and everolimus-exemestane (EVE-EXE) for second line hormonal treatment (HT) of metastatic breast cancer (MBC) with lobular histology: A propensity score matched analysis | 2020 | Duplicate |
| Orlandi, A., Iattoni, E., Pizzuti, L., Fabbri, A., Botticelli, A., Di Dio, C., Palazzo, A., Garufi, G., Indellicati, G., Alesini, D., Carbognin, L., Paris, I., Vaccaro, A., Moscetti, L., Fabi, A., Magri, V., Naso, G., Cassano, A., Vici, P., Giannarelli, D., Franceschini, G., Marchetti, P., Bria, E., Tortora, G. | Palbociclib plus fulvestrant or everolimus plus exemestane for pretreated advanced breast cancer with lobular histotype in er+/her2- patients: A propensity score-matched analysis of a multicenter retrospective patient series | 2020 | Population - mixed patient population or unclear whether population has failed CDK4/6i |
| Orlandi, A., Iattoni, E., Pizzuti, L., Fabbri, M. A., Botticelli, A., Di Dio, C., Palazzo, A., Garufi, G., Indellicati, G., Alesini, D., Carbognin, L., Paris, I., Vaccaro, A., Moscetti, L., Cassano, A., Vici, P., Giannarelli, D., Marchetti, P., Bria, E., Tortora, G. | Palbociclib-fulvestrant (PALBO-FUL) and everolimus-exemestane (EVEEXE) for second line hormonal treatment (HT) of metastatic breast cancer (MBC) with lobular histology: A propensity score matched analysis of a multicenter 'real-world' patients (pts) series | 2019 | Duplicate |
| Orlandi, Armando, Aroldi, Francesca, Garutti, Mattia, Di Dio, Carmela, Garufi, Giovanna, Iattoni, Elena, Palazzo, Antonella, Indellicati, Giulia, Franceschini, Gianluca, Cassano, Alessandra, Bria, Emilio, Tortora, Giampaolo | Poor efficacy of palbociclib in second-line treatment of metastatic lobular breast cancer in a case series: Use before or never more? | 2020 | Population - mixed patient population or unclear whether population has failed CDK4/6i |
| Orringer, D. A., Chen, T., Pastore, S., Darvishian, F., Toth, H., Schnabel, F. | Genomic heterogeneity and associated clinical outcomes of breast cancers treated with CDK4/6 inhibitors: Insights from real-world clinical genomic data | 2021 | Population - mixed patient population or unclear whether population has failed CDK4/6i |
| O'Shaughnessy, J., Paulson, S., Brisbin, L., Lindsey, J., Williford, A., Lisi, M., Parikh, R. C., Simmons, S., Balu, S. | A real-world assessment of PIK3CA testing and a l pel isi b treatment patterns among metastatic breast cancer patients in a community oncology setting | 2022 | Population - mixed patient population or unclear whether population has failed CDK4/6i |
| O'Shaughnessy, J., Woeckel, A., Pistilli, B., Hegg, R., Vahdat, L. T., Vuina, D., Asad, Z. V. K. P., Smith, T. W., Kim, J., Krop, I. | Clinical outcomes with alpelisib (ALP) plus fulvestrant (FUL) after prior treatment (tx) with FUL in patients (pts) with advanced breast cancer (ABC): A real-world (RW) analysis | 2022 | Population - failed CDK4/6i but not in 1L |
| Palumbo, R., Torrisi, R., Quaquarini, E., Sottotetti, F., Gambaro, A., Collova, E., Ferzi, A., Fava, S., Agostinetto, E., Tagliaferri, B., Licata, L., Teragni, C., Bernardo, A. | Patterns of treatment and outcome of palbociclib plus endocrine therapy in hormone receptor-positive (HR+)/HER2 receptor-negative (HER2-) metastatic breast cancer (MBC): A real life multicenter Italian study | 2019 | Duplicate |
| Palumbo, R., Torrisi, R., Sottotetti, F., Presti, D., Rita Gambaro, A., Collova, E., Ferzi, A., Agostinetto, E., Maria Teragni, C., Saltalamacchia, G., Tagliaferri, B., Balletti, E., Bernardo, A., Quaquarini, E. | Patterns of treatment and outcome of palbociclib plus endocrine therapy in hormone receptor-positive/HER2 receptor-negative metastatic breast cancer: a real-world multicentre Italian study | 2021 | Outcome (e.g., only reports patient-reported outcomes/utilities) |
| Pancirov, M., Majic, A., Tomic, S., Dolic, K., Krnic, M., Petric-Mise, B., Vrdoljak, E. | First experiences of alpelisib treatment of HR + HER2-metastatic breast carcinoma in Croatia | 2020 | Population - mixed patient population or unclear whether population has failed CDK4/6i |
| Patt, D., Liu, X., Li, B., McRoy, L., Layman, R. M., Brufsky, A. | Real-World Treatment Patterns and Outcomes of Palbociclib Plus an Aromatase Inhibitor for Metastatic Breast Cancer: Flatiron Database Analysis | 2022 | Population - mixed patient population or unclear whether population has failed CDK4/6i |
| Perez-Vargas, J. C. S., Cudos, A. G., Francia, V. M. R., Garrido-Lecca, A. L., Lizaraso, S. F., Murillo, S. M. | REAL WORLD DATA OF CYCLIN-DEPENDENT KINASE 4/6 INHIBITORS IN A EUROPEAN AND LATIN-AMERICAN LUMINAL ADVANCED BREAST CANCER POPULATION. ANALYSIS OF TWO CENTERS | 2019 | Population - failed CDK4/6i but not in 1L |
| Petracci, F., Abuin, G. G., Pini, A., Chacon, M. | RENATA study-Latin American prospective experience: Clinical outcome of patients treated with palbociclib in hormone receptor-positive metastatic breast cancer-real-world use | 2020 | Population - mixed patient population or unclear whether population has failed CDK4/6i |
| Pizzuti, L., Giordano, A., Michelotti, A., Mazzotta, M., Natoli, C., Gamucci, T., De Angelis, C., Landucci, E., Diodati, L., Iezzi, L., Mentuccia, L., Fabbri, A., Barba, M., Sanguineti, G., Marchetti, P., Tomao, S., Mariani, L., Paris, I., Lorusso, V., Vallarelli, S., Cassano, A., Airoldi, F., Orlandi, A., Moscetti, L., Sergi, D., Sarobba, M. G., Tonini, G., Santini, D., Sini, V., Veltri, E., Vaccaro, A., Ferrari, L., De Tursi, M., Tinari, N., Grassadonia, A., Greco, F., Botticelli, A., La Verde, N., Zamagni, C., Rubino, D., Cortesi, E., Magri, V., Pomati, G., Scagnoli, S., Capomolla, E., Kayal, R., Scinto, A. F., Corsi, D., Cazzaniga, M., Laudadio, L., Forciniti, S., Mancini, M., Carbognin, L., Seminara, P., Barni, S., Samaritani, R., Roselli, M., Portarena, I., Russo, A., Ficorella, C., Cannita, K., Carpano, S., Pistelli, M., Berardi, R., De Maria, R., Sperduti, I., Ciliberto, G., Vici, P. | Palbociclib plus endocrine therapy in HER2 negative, hormonal receptor-positive, advanced breast cancer: A real-world experience | 2019 | Population - mixed patient population or unclear whether population has failed CDK4/6i |
| Princic, N., Aizer, A., Tang, D. H., Smith, D. M., Johnson, W., Bardia, A. | Predictors of systemic therapy sequences following a CDK 4/6 inhibitor-based regimen in post-menopausal women with hormone receptor positive, HEGFR-2 negative metastatic breast cancer | 2019 | Outcome (e.g., only reports patient-reported outcomes/utilities) |
| Raghavendra, A. S., Ha, M. J., Kettner, N. M., Damodaran, S., Layman, R., Hunt, K. K., Shen, Y., Tripathy, D., Keyomarsi, K. | Pa l bocicl i b plus endocrine therapy significantly enhances overall survival of HR+/HER2-metastatic breast cancer patients compared to endocrine therapy alone-A large institutional study | 2022 | Population - mixed patient population or unclear whether population has failed CDK4/6i |
| Ramos Rodriguez, J., Hernandez Rojas, S., Gonzalez Perera, I., Vina Romero, M. M., Nazco Casariego, G. J., Merino Alonso, F. J., Garcia Gil, S., Del Rosario Garcia, B., Cantarelli, L., Gutierrez Nicolas, F. | Cyclin dependent kinases 4/6 inhibitors: New options in HR+ HER2-breast cancer | 2019 | Population - mixed patient population or unclear whether population has failed CDK4/6i |
| Raphael, A., Salmon-Divon, M., Epstein, J., Zahavi, T., Sonnenblick, A., Shachar, S. S. | Alpelisib Efficacy in Hormone Receptor-Positive HER2-Negative PIK3CA-Mutant Advanced Breast Cancer Post-Everolimus Treatment | 2022 | Population - failed CDK4/6i but not in 1L |
| Rapti, K., Papadimitraki, E., Konstantis, A., Raja, F., Spurrell, E., Benafif, S., Roylance, R. | HR+/HER2-advanced breast cancer: Real world use of abemaciclib in UCLH (university College London Hospital) breast unit | 2020 | Population - mixed patient population or unclear whether population has failed CDK4/6i |
| Rath, S., Elamarthi, P., Parab, P., Gulia, S., Nandhana, R., Mokal, S., Kembhavi, Y., Perumal, P., Bajpai, J., Ghosh, J., Gupta, S. | Efficacy and safety of palbociclib and ribociclib in patients with estrogen and/or progesterone receptor positive, HER2 receptor negative metastatic breast cancer in routine clinical practice | 2021 | Population - failed CDK4/6i but not in 1L |
| Rinnerthaler, G., Gampenrieder, S. P., Tinchon, C., Petzer, A. L., Suppan, C., Heibl, S., Voskova, D., Zabernigg, A. F., Egle, D., Sandholzer, M., Singer, C. F., Roitner, F., Andel, J., Hubalek, M., Knauer, M., Greil, R. | First-line treatment of hormonereceptor positive metastatic breast cancer (MBC) ineveryday practice: Results from the AustrianAGMT-MBC-Registry | 2021 | Population - mixed patient population or unclear whether population has failed CDK4/6i |
| Rodriguez, B., Zepeda, L. L., Noguez-Ramos, A., Juarez, D. V., Limas, C. P. C., Porras, G. O. R., Salcedo, I., Rivera, S., Aguayo, A., Vazquez, Y. C. B., Galindo, A. A. L., Gerson, D. S., Perez-Zincer, F., Serrano, J. A., Martinez-Herrera, J. F., Olivares, G., Villalobos, A., De Leon, C. G., Gerson, R. | Real-world clinical outcomes in patients receiving cyclindependent kinase 4/6 inhibitors (iCDK 4/6) for hormone receptorpositive, human epidermal growth factor receptor 2-negative (HR+/HER2-) metastatic breast cancer in Mexico | 2020 | Population - mixed patient population or unclear whether population has failed CDK4/6i |
| Rodriguez, P. B., Ferrer, J. S., Lison, L. C. F., Hernandez, C. C., Alejandre, P. T., Aguilar, J. J. D. | Evaluation of palbociclib induced neutropenia in breast cancer | 2021 | Population - mixed patient population or unclear whether population has failed CDK4/6i |
| Rozenblit, M., Pusztai, L., Adelson, K., Mougalian, S. | Patterns of treatment with everolimus and exemestane in hormone receptor positive HER2 negative metastatic breast cancer in the era of targeted therapy | 2020 | Duplicate |
| Rugo, H. S., Dieras, V., Cortes, J., Patt, D., Wildiers, H., O'Shaughnessy, J., Zamora, E., Yardley, D. A., Carter, G. C., Sheffield, K. M., Li, L., Andre, V. A. M., Li, X. I., Frenzel, M., Huang, Y. J., Dickler, M. N., Tolaney, S. M. | Real-world survival outcomes of heavily pretreated patients with refractory HR+, HER2-metastatic breast cancer receiving single-agent chemotherapy-a comparison with MONARCH 1 | 2020 | Population - all other reasons |
| Sampedro-Gimeno, T., Pampin-Sanchez, R., Barbazan-Vazquez, F. J., Reguero-Cuervo, V., Galeazzi-Martinez, V., Pelaez-Fernandez, I. | Observational real world data with palbociclib associated to hormone therapy for advanced breast carcinoma | 2021 | Population - all other reasons |
| Savva-Bordalo, J., Cunha, J., Monteiro, S., Redondo, P., Sousa, S., Pereira, D. | PO68 REAL WORLD EFFECTIVENESS AND QUALITY OF LIFE OF RIBOCICLIB AND LETROZOLE IN ADVANCED BREAST CANCER | 2021 | Population - mixed patient population or unclear whether population has failed CDK4/6i |
| Schneeweiss, A., Ettl, J., Luftner, D., Beckmann, M. W., Belleville, E., Fasching, P. A., Fehm, T. N., Geberth, M., Haberle, L., Hadji, P., Hartkopf, A. D., Hielscher, C., Huober, J., Ruckhaberle, E., Janni, W., Kolberg, H. C., Kurbacher, C. M., Klein, E., Lux, M. P., Muller, V., Nabieva, N., Overkamp, F., Tesch, H., Laakmann, E., Taran, F. A., Seitz, J., Thomssen, C., Untch, M., Wimberger, P., Wuerstlein, R., Volz, B., Wallwiener, D., Wallwiener, M., Brucker, S. Y. | Initial experience with CDK4/6 inhibitor-based therapies compared to antihormone monotherapies in routine clinical use in patients with hormone receptor positive, HER2 negative breast cancer - Data from the PRAEGNANT research network for the first 2 years of drug availability in Germany | 2020 | Population - mixed patient population or unclear whether population has failed CDK4/6i |
| Seki, H., Sakurai, T., Sakurada, A., Kinoshita, T., Shimizu, K. | Subsequent-abemaciclib Treatment After Disease Progression on Palbociclib in Patients With ER-positive HER2-negative Metastatic Breast Cancer | 2022 | Population - failed CDK4/6i but not in 1L |
| Sendur, M. A. N., Cakar, B., Hizal, M., Eraslan, E., Aksoy, S., Paydas, S., Demir, N., Sen, F., Bulut, G., Oruc, K., Oyan Uluc, B., Ozdemir, N., Sakin, A., Erdem, D., Ozkan, M., Disel, U., Ekinci, F., Okten, I. N., Ozer, L., Gokmen, E. | Talazoparib in locally advanced or metastatic breast cancer patients: experience from an early access program in Turkey | 2021 | Population - mixed patient population or unclear whether population has failed CDK4/6i |
| Shen, L., Zhou, J., Chen, Y., Ding, J., Wei, H., Liu, J., Xia, W., Xie, B., Xie, X., Li, X., Dai, Y., Zhang, G., Qiu, X., Li, C., Sun, S., Chen, W., Gong, D., Li, H., Huang, J., Jiang, X., Ni, C. | Treatment patterns, effectiveness, and patient-reported outcomes of palbociclib therapy in Chinese patients with advanced breast cancer: A multicenter ambispective real-world study | 2022 | Population - mixed patient population or unclear whether population has failed CDK4/6i |
| Shen, X. B., Li, G. L., Zheng, Y. B., Chen, Z. H., Cao, W. M., Shao, X. Y., Wang, X. J. | Everolimus combined with endocrine therapy in advanced HR-positive, HER2-negative Chinese breast cancer patients: A retrospective study | 2021 | Population - mixed patient population or unclear whether population has failed CDK4/6i |
| Sinjari, M., Brandi, M., Ceddia, S., Cosimati, A., Verrico, M., Gozzi, E., Mannino, A., Di Lisa, F. S., Rossi, L., Tomao, S. | Real life incidence and management of adverse events (AES) in women with estrogenreceptor (HR)-positive and (HER2)-negative advanced breast cancer treated with palbociclib: A single institution experience | 2019 | Population - mixed patient population or unclear whether population has failed CDK4/6i |
| Smyth, E. N., Beyrer, J., Saverno, K. R., Hadden, E., Abedtash, H., DeLuca, A., Lawrence, G. W., Rybowski, S. | Real-World Patient Characteristics, Utilization Patterns, and Outcomes of US Patients with HR+, HER2- Metastatic Breast Cancer Treated with Abemaciclib | 2022 | Population - failed CDK4/6i but not in 1L |
| Tamragouri, K., Cobleigh, M. A., Rao, R. D. | Abemaciclib with or without fulvestrant for the treatment of hormone receptor-positive and HER2-negative metastatic breast cancer with disease progression following prior treatment with palbociclib | 2019 | Population - failed CDK4/6i but not in 1L |
| Terada, M., Masataka, S., Masaya, H., Akiyo, Y., Naomi, G., Haruru, K., Yayoi, A., Ayumi, K., Kayoko, S., Nanae, H., Makiko, M., Yuri, O., Hiroji, I. | Palbociclib in clinical use for metastatic breast cancer at a single institution | 2019 | Population - mixed patient population or unclear whether population has failed CDK4/6i |
| Tolosa Ortega, P., Cejalvo, J. M., Moragon Terencio, S., Carril-Ajuria, L., Bermejo, B., Ruiz, A., Hernando Melia, C., Sanchez-Torre, A., Martinez, M. T., Herrera, M., Gambardella, V., Lema, L., Roda, D., Bernal, E., Rentero-Garrido, P., Lluch, A., Ciruelos, E. M., Cervantes, A., Manso, L. | 57P Benefit of CDK4/6 inhibitors beyond PIK3CA mutations in metastatic breast cancer patients | 2020 | Population - mixed patient population or unclear whether population has failed CDK4/6i |
| Tripathy, D., Rocque, G., Blum, J. L., Karuturi, M. S., McCune, S., Kurian, S., Moezi, M. M., Anderson, D., Gauthier, E., Zhang, Z., Montelongo, M., Wang, Y. | Real-world clinical outcomes of palbociclib plus endocrine therapy (ET) in hormone receptor-positive advanced breast cancer: Results from the POLARIS trial | 2022 | Population - mixed patient population or unclear whether population has failed CDK4/6i |
| Trocio, J., Lin, J., Fisher, M. D., Hu, N., Davis, C., McRoy, L., Walker, M. S., Iyer, S. | Real-world treatment patterns and clinical outcomes with palbociclib combination therapy received in US community oncology practices | 2019 | Population - mixed patient population or unclear whether population has failed CDK4/6i |
| Tryfonopoulos, D., Tzouda, V., Zouki, D., Kyriakidou, A., Sarris, E., Grivas, A., Gouveris, P., Papaxoinis, G., Demiri, S. G. | Single center experience with alpelisib-fulvestrant in heavily pretreated women with HR(+), HER-2 (-) metastatic breast cancer | 2022 | Population - mixed patient population or unclear whether population has failed CDK4/6i |
| Turner, S., Chia, S. K. L., Kanakamedala, H., Hsu, W. C., Park, J., Chandiwana, D., Ridolfi, A., Yu, C. L., Zarate, J. P., Rugo, H. S. | Real-world effectiveness of alpelisib (ALP) + fulvestrant (FUL) compared with standard treatment among patients (Pts) with hormone-receptor positive (HR+) human epidermal growth factor receptor-2 negative (HER2-) PIK3CA-mutated (Mut) advanced breast cancer (ABC) | 2020 | Population - failed CDK4/6i but not in 1L |
| Untch, M., Fasching, P. A., McLaurin, K. | A real-world evidence study of CDK4/6 inhibitor treatment patterns and outcomes in metastatic breast cancer by gbrcamutation status | 2020 | Population - mixed patient population or unclear whether population has failed CDK4/6i |
| Urch, K., Levarda, N. P., Jazvic, M., Soldic, Z., Frobe, A. | Treatment with CDK 4/6 inhibitors in metastatic hormon receptor positive, HER-2 negative breast cancer-a single center experience | 2020 | Population - mixed patient population or unclear whether population has failed CDK4/6i |
| Varella, L., Eziokwu, A. S., Jia, X., Kruse, M., Moore, H. C. F., Budd, G. T., Abraham, J., Montero, A. J. | Real-world clinical outcomes and toxicity in metastatic breast cancer patients treated with palbociclib and endocrine therapy | 2019 | Population - mixed patient population or unclear whether population has failed CDK4/6i |
| Visani, L., Ratosa, I., Scoccimarro, E., Becherini, C., Saieva, C., Desideri, I., Scotti, V., Ozarem, M., Ribnikar, D., Aquilano, M., Cerbai, C., Orzalesi, L., Bernini, M., Sanchez, L., Nori, J., Bianchi, S., Meattini, I., Livi, L. | Safety and efficacy of concomitant radiation and CDK4/6 inhibitors in breast cancer patients | 2021 | Population - mixed patient population or unclear whether population has failed CDK4/6i |
| Waks, A. G., Gharaibeh, M., Sjekloca, N., Poluparthi, N., Shah, A., Bergamaco, E., MacCannell, T., Leung, G., Ntalla, I., Oko-osi, H., Tolaney, S. M. | Unmet need in heavily pre-treated patients with HR+/HER2- metastatic breast cancer (mBC) in the US: A ConcertAI analysis | 2022 | Population - failed CDK4/6i but not in 1L |
| Wander, S. A., Spring, L. M., Stein, C. R., Yuen, M., Zangardi, M., O'Shaughnessy, J., Bardia, A. | Abemaciclib after prior palbociclib exposure in patients with metastatic hormone-receptor positive (HR+)/HER2-breast cancer | 2019 | Population - failed CDK4/6i but not in 1L |
| Wander, S. A., Weipert, C., Liao, J., Zhang, N., Razavi, P. | Use of real-world data (RWD) to assess the utility of cell-free circulating tumor DNA (cfDNA) in identifying resistance to early treatment in advanced breast cancer (aBC) | 2022 | Population - mixed patient population or unclear whether population has failed CDK4/6i |
| Wander, S. A., Zangardi, M., Niemierko, A., Kambadakone, A., Kim, L. S. L., Xi, J., Pandey, A. K., Spring, L., Stein, C., Juric, D., Kuter, I., Moy, B., Mulvey, T. M., Vidula, N., Isakoff, S. J., Yuen, M., Brufsky, A., Ma, C. X., O'Shaughnessy, J., Bardia, A. | A multicenter analysis of abemaciclib after progression on palbociclib in patients (pts) with hormone receptor-positive (HR+)/HER2-metastatic breast cancer (MBC) | 2019 | Population - failed CDK4/6i but not in 1L |
| Wang, R., Yang, Y., Ye, W. W., Xiang, J., Chen, S., Zou, W. B., Wang, X. J., Chen, T., Cao, W. M. | Case Report: Significant Response to Immune Checkpoint Inhibitor Camrelizumab in a Heavily Pretreated Advanced ER+/HER2- Breast Cancer Patient With High Tumor Mutational Burden | 2020 | Population - failed CDK4/6i but not in 1L |
| Wang, X., Ascha, M., Green, T., Lewin, H. G., Showalter, T. N., Goldstein, L. J., Calip, G. S., Whitaker, K. D. | Racial disparities in second-line (2L) treatment and overall survival among patients (pts) with hormone receptor positive HER2 negative (HR+HER2-) metastatic breast cancer (mBC) treated in routine practice | 2021 | Population - mixed patient population or unclear whether population has failed CDK4/6i |
| Watanabe, K., Kuwahara, S., Tachikawa, H., Maeda, H., Yamamoto, M., Tomioka, N., Takahashi, M. | P66-6 Efficacy of everolimus in HR+ HER2- metastatic breast cancer patients after progression on CDK4/6 inhibitors | 2022 | Population - failed CDK4/6i but not in 1L |
| Watanabe, K., Niikura, N., Kikawa, Y., Oba, M., Kobayashi, K., Tada, H., Ozaki, S., Toh, U., Yamamoto, Y., Tsuneizumi, M., Okuno, T., Iwakuma, N., Takeshita, T., Iwamoto, T., Ishiguro, H., Masuda, N., Saji, S. | Fulvestrant with additional palbociclib in advanced or metastatic hormone receptor-positive HER2-negative breast cancer after progression to fulvestrant monotherapy: JBCRG- M07 (FUTURE trial) | 2022 | Population - mixed patient population or unclear whether population has failed CDK4/6i |
| West, M., Kaempf, A., Goodyear, S., Kartika, T., Ribkoff, J., Mitri, Z. I. | Real-world analysis of disease progression after CDK 4/6 inhibitor (CDKi) therapy in patients with hormone receptor positive (HR+)/HER2-metastatic breast cancer (MBC) | 2021 | Population - failed CDK4/6i but not in 1L |
| Whitaker, K. D., Wang, X., Ascha, M., Showalter, T. N., Lewin, H. G., Calip, G. S., Goldstein, L. J. | Racial inequities in second-line treatment and overall survival among patients with metastatic breast cancer | 2022 | Population - mixed patient population or unclear whether population has failed CDK4/6i |
| Wockel, A., Fasching, P. A., Guderian, G., Heim, J., Jackisch, C., Luck, H. J., Luftner, D., Marme, F., Reimer, T., Decker, T. | RIBANNA-Real-world evidence of ribociclib plus aromatase inhibitor, or endocrine monotherapy, or chemotherapy as first-line therapy for postmenopausal women with HR+, HER2-advanced breast cancer (aBC) | 2019 | Incomplete/Insufficient/Partial data |
| Yang, Y., Sun, C., Huang, X., Zeng, T., Hua, Y., Yang, F., Li, W., Yin, Y. | 56P Treatment of palbociclib in hormone receptor-positive breast cancer in China: A real-world study | 2020 | Population - mixed patient population or unclear whether population has failed CDK4/6i |
| Yildirim, H. C., Mutlu, E., Chalabiyev, E., Ozen, M., Keskinkilic, M., On, S., Celebi, A., Dursun, B., Acar, O., Kahraman, S., Aykan, M. B., Kaman, O., Dogan, A., Erdogan, A. P., Melisa Celayir, O., Gunenc, D., Guven, D. C., Vedat Bayoglu, I., Yavuzsen, T., Hacibekiroglu, I., Inanc, M., Kilickap, S., Yalcin, S., Aksoy, S. | Clinical outcomes of cyclin-dependent kinase 4-6 (CDK 4-6) inhibitors in patients with male breast cancer: A multicenter study | 2022 | Population - mixed patient population or unclear whether population has failed CDK4/6i |
| Zattarin, E., Fabbroni, C., Ligorio, F., Marra, A., Corti, C., Bernocchi, O., Sirico, M., Generali, D. G., Curigliano, G., Bianchi, G., Capri, G., Rivoltini, L., De Braud, F. G. M., Vernieri, C. | Association between the neutrophil-to-lymphocyte and platelet-to-lymphocyte ratios and efficacy of CDK 4/6 inhibitors in advanced breast cancer: The observational multicenter Italian PALMARES study | 2020 | Population - mixed patient population or unclear whether population has failed CDK4/6i |
| Zhang, L., Song, G., Shao, B., Xu, L., Xiao, Y., Wang, M., Sumou, I. K., Zhang, Y., Liang, X., Jiang, H., Li, H. | The efficacy and safety of palbociclib combined with endocrine therapy in patients with hormone receptor-positive HER2-negative advanced breast cancer: a multi-center retrospective analysis | 2022 | Population - mixed patient population or unclear whether population has failed CDK4/6i |
| Zhang, L., Wang, H., Yang, J., Lv, Z., Zhou, L., Zhang, H., Ji, X., Zhao, J., Song, C., Xu, L., Zhao, S., Shao, Y., Liu, J., Li, M. | Clinical outcomes and clinical/genetic risk factors of palbociclib plus endocrine therapy (ET) for HR+HER2- advanced breast cancer (ABC) patients in Chinese multicenter study of real-world practices | 2022 | Population - mixed patient population or unclear whether population has failed CDK4/6i |
| Zhang, Y., Chen, W., Chen, S., Yang, Q., Ouyang, Z. | Early Application of Palbociclib Plus Endocrine Therapy in HR+/HER2- Metastatic Breast Cancer: A Better Choice Based on Data From the Chinese Population | 2022 | Population - mixed patient population or unclear whether population has failed CDK4/6i |
| Zhong, B., Zhang, J., Wu, J., Sun, L., Li, S., Zeng, X., Gan, L. | Efficacy and safety of palbociclib plus endocrine therapy for patients with HR+/HER2- advanced breast cancer in real-world clinical practice | 2022 | Population - failed CDK4/6i but not in 1L |
| Zhou, J., Wu, X., Zhang, H., Wang, X., Yuan, Y., Zhang, S., Jiang, Z., Wang, T. | Clinical outcomes of tucidinostat-based therapy after prior CDK4/6 inhibitor progression in hormone receptor-positive heavily pretreated metastatic breast cancer | 2022 | Population - failed CDK4/6i but not in 1L |
|  | Women With Advanced Breast Cancer | 2020 | Study design |
| Wander, S. A., Han, H. S., Zangardi, M. L., Niemierko, A., Mariotti, V., Kim, L. S. L., Xi, J., Pandey, A., Dunne, S., Nasrazadani, A., Kambadakone, A., Stein, C., Lloyd, M. R., Yuen, M., Spring, L. M., Juric, D., Kuter, I., Sanidas, I., Moy, B., Mulvey, T., Vidula, N., Dyson, N. J., Ellisen, L. W., Isakoff, S., Wagle, N., Brufsky, A., Kalinsky, K., Ma, C. X., O'Shaughnessy, J., Bardia, A. | Clinical Outcomes With Abemaciclib After Prior CDK4/6 Inhibitor Progression in Breast Cancer: A Multicenter Experience | 2021 | Population - mixed patient population or unclear whether population has failed CDK4/6i |
