# Supplementary material for: Systematic literature review of real-world evidence for treatments in HR+/HER2- second-line LABC/mBC after first-line treatment with CDK4/6i
Source: BMC Cancer. 2024 May 23;24:631. doi: 10.1186/s12885-024-12269-8 (PMC11112888; doi:10.1186/s12885-024-12269-8)
Supplement: Supplementary file 2 — Supplementary Material 2 [file 12885_2024_12269_MOESM2_ESM.docx]

Table S2. Quality assessment for full-text real-world evidence articles

|  | | **Q1: Representativeness of the exposed cohort** | | **Q2: Selection of the nonexposed cohort** | | **Q3: Ascertainment of exposure** | | **Q4: Outcome of interest not present at start of study** | | **Q1: Comparability** | | | **Q1: Assessment of outcome** | | **Q2: Was follow-up long enough for outcomes to occur?** (*12 months follow-up deemed adequate*) | | | **Q3: Adequacy of follow-up** *(>10% lost to follow-up = inadequate rate of follow-up for RWE studies)* | | **Total** |
| --- | --- | --- | --- | --- | --- | --- | --- | --- | --- | --- | --- | --- | --- | --- | --- | --- | --- | --- | --- | --- |
|  |  |  |  |  |  |  |  |  |  | **(2 stars available)** | | |  |  |  |  |  |  |  |  |
| **Author** | **Year** | **Response** | **Star (X)** | **Response** | **Star (X)** | **Response** | **Star (X)** | **Response** | **Star (X)** | **Response** | **Star (X)** | **Star (X)** | **Response** | **Star (X)** | **Response** | **Star (X)** | **Duration of follow-up and rationale for assessment** | **Response** | **Star (X)** | **Total: (/9)** |
| Martin | 2022 | a) truly representative of the average HR+/HER2- mBC patient treated with a first-line CDK4/6i in the community | X | NA |  | a) secure record | X | a) Yes | X | NA |  |  | a) independent assessment | X | a) Yes | X | the median OS = 35.7 months | d) no statement |  | **5** |
| Endo | 2022 | b) somewhat representative of the average HR+/HER2- mBC patient treated with a first-line CDK4/6i in the community | X | a) drawn from the same community as the exposed cohort | X | a) secure record | X | a) Yes | X | NR |  |  | a) independent assessment | X | a) Yes | X | The median length of follow-up was 13.8 months (9.8–43.6 months) for all patients in the CDK4/6i (first) group, 27.5 months (0.5–63.4 months) in the CDK4/6i (second) group, and 30.3 months (1.4–117.0 months) in the non-CDK4/6i group. | d) no statement |  | **6** |
| Choong | 2022 | a) truly representative of the average HR+/HER2- mBC patient treated with a first-line CDK4/6i in the community | X | NA |  | a) secure record | X | a) Yes | X | NA |  |  | a) independent assessment | X | a) Yes | X | Median follow-up in the 1L cohort was 40.5 months (Q1–Q3 30.3–48.9 months) and median post-CDK4/6i follow-up was 25.5 months (Q1–Q3 12.1–35.3 months). | d) no statement |  | **5** |
| Rozenblit | 2022 | a) truly representative of the average HR+/HER2- mBC patient in the community | X | NA |  | a) secure record | X | a) Yes | X | NA |  |  | a) independent assessment | X | a) Yes | X | mOS=21.8 months | d) no statement |  | **5** |
| Li | 2021 | b) somewhat representative of the average HR+/HER2- mBC patient in the community | X | NA |  | a) secure record | X | a) Yes | X | NA |  |  | a) independent assessment | X | b) No |  | median follow-up of 9.2 (range, 3.8–35.4) | d) no statement |  | **4** |
| Amaro | 2021 | a) truly representative of the average HR+/HER2- mBC patient in the community | X | NA |  | a) secure record | X | a) Yes | X | NA |  |  | a) independent assessment | X | a) Yes | X | The median follow-up time from advanced diagnosis was 28.1 months | d) no statement |  | **5** |
| Xi | 2019 | a) truly representative of the average HR+/HER2- mBC patient treated with a first-line CDK4/6i in the community | X | NA |  | a) secure record | X | a) Yes | X | NA |  |  | a) independent assessment | X | a) Yes | X | median follow-up of 19.5 months (range, 1.7–34.9 months) | d) no statement |  | **5** |
| Mougalian | 2019 | b) somewhat representative of the average HR+/HER2- mBC patient in the community | X | NA |  | a) secure record | X | a) Yes | X | NA |  |  | a) independent assessment | X | a) Yes | X | Mean (SD) of follow up from initiation of 1L therapy: 24.3 (8.7) | d) no statement |  | **5** |
| Bashour | 2017 | a) somewhat representative of the average HR+/HER2- mBC patient in the community | X | NA |  | a) secure record | X | a) Yes | X | NA |  |  | a) independent assessment | X | a) Yes | X | Median follow-up = 11.65 (~12) | d) no statement |  | **5** |
| Karacin | 2023 | a) truly representative of the average HR+/HER2- mBC patient in the community | X | NA |  | a) secure record | X | a) Yes | X | NA |  |  | a) independent assessment | X | a) Yes | X | Median duration of CDKi for group of interest = 15 months (9-46) | d) no statement |  | **5** |

**Table S3. Study and patient characteristics across included real-world evidence studies**

| Study Author, Year | Publication type | Study design | Country | Data collection period | Sample size | Age^a^ (years), median (interquartile) | Follow-up duration of entire patient cohort, median (interquartile) (months) | Follow-up duration of patients treated with CDK4/6i | 1L regimen of patients failed CDK4/6i | 2L regimen of patients failed CDK4/6i |
| --- | --- | --- | --- | --- | --- | --- | --- | --- | --- | --- |
| Moscetti, 2022 | Conference abstract | Observational retrospective/ prospective | Italy | NR | 26 | NR | NR | NR | NR | AI n=1 (3.8%) Fulvestrant n=2 (7.7%) Chemotherapy n=20 (76.9%) Everolimus n=3 (11.5%) |
| Menichetti, 2022 | Conference abstract | Observational multi-institution retrospective/ prospective | Italy | Oct 2015 - Jan 2021 | NR | NR | 31 | NR | NR | NR |
| Martin, 2022 | Full-text article | Retrospective | US | 2015 - 2020 | 839 | 64.4 (28-84) | NR | NR | NR | AI n=23 (2.7%) CDK4/6i n=4 (0.5%) CDK4/6i+AI n=97 (11.6%) CDK4/6i+fulvestrant n=160 (19.1%) CDK4/6i+fulvestrant + AI n=35 (4.2%) CDK4/6i+fulvestrant+tamoxifen n=3 (0.4%) CDK4/6i+tamoxifen n=3 (0.4%) Chemotherapy n=249 (29.7%) Fulvestrant n=70 (8.3%) Fulvestrant+AI n=14 (1.7%) Everolimus n=99 (11.7%) PARP Inhibitor n=4 (0.5%) Alpelisib n=16 (1.9%) Tamoxifen n=11 (1.3%) Unspecified n=51 (6.1%) |
| Marschner, 2022 | Conference abstract | Prospective registry | Germany | Jan 2018 - Aug 2021 | 113 | 64 (NR) | NR | NR | NR | Chemotherapy n=79 (70.0%) ET n=22 (20.0%) CDK4/6i n=10 (9.0%) PARPi n= 2 (2.0%) |
| Kalinsky, 2022 | Conference abstract | Retrospective | US | Nov 1995 - Oct 2020 | 259 | 61 (26-86) | NR | NR | NR | CDK4/6i n=165 (63.7%) non-CDK4/6i n=94 (36.3%) |
| Gousis, 2022 | Conference abstract | Single-institution retrospective review | UK | Feb 2020 - Feb 2021 | 38 | NR | NR | NR | CDK4/6i + AI | Chemotherapy n=22 (22.6%) Fulvestrant n=7 (18.4%) Tamoxifen n=1 (2.6%) Everolimus+exemestane n=4 (10.5%) Unspecified n=4 (10.5%) |
| Endo, 2022 | Full-text article | Retrospective chart review | Japan | Jan 2016 - Dec 2020 | 14 | 1L CDK4/6: 51 (34–78) 2L CDK 4/6: 57 (35–81) non-CDK4/6i: 63 (27–88) | 1L CDK4/6i: 13.8 (9.8-43.6) 2L CDK4/6i: 27.5 (0.5–63.4) non-CDK4/6i: 30.3 (1.4–117.0) | NR | NR | Everolimus+exemestane n=3 (21.4%) CDK4/6i n=2 (14.3%) Chemotherapy n=5 (35.7%) Endocrine monotherapy n=28.6%) |
| Choong, 2022 | Full-text article | Retrospective | US | Dec 2014 - Sep 2020 | 66 | 59 (50-66) | 1L: 40.5 (30.3-48.9) 2L: 35.8 (12.1-35.3) | NR | Palbociclib/letrozole  n=74 (81.3%) Palbociclib/fulvestrant n=11 (12.1%) Palbociclib/exemestane n=3 (3.3%) Palbociclib/anastrozole n=3 (3.3%) | Palbociclib/fulvestrant n=5 (7.6%) Abemaciclib n=2 (3.0%) Letrozole n=3 (5.1%) Fulvestrant n=15 (22.7%) Tamoxifen/Endoxifen n=2 (3.0%) Exemestane n=2 (3.0%) Everolimus/exemestane n=17 (25.8%) Everolimus/fulvestrant n=1 (1.5%) Alpelisib/fulvestrant n=4 (6.1%) Chemotherapy n=14 (21.2%) Unspecified n=1 (1.5%) |
| Rozenblit, 2021 | Full-text article | Retrospective analysis of electronic health record-derived data | US | 2012 - 2018 | 54 | <60: 202 (32.5%) ≥60: 420 (67.5%) | NR | NR | ET+CDK 4/6i n=54 (100%) | Everolimus+exemestane n=54 (100%) |
| Li, 2021 | Full-text article | Retrospective, observational multicenter study | China | Aug 2017 - Apr 2020 | 35 | 55 (28–82) | 9.2 (3.8 - 35.4) | NR | Palbociclib+ET n=35 (100%) | Chemotherapy n=22 (62.9%) ET n=13 (37.1%) |
| Amaro, 2021 | Full-text article | Retrospective, population-based cohort study | Canada | Jan 2016 - Jun 2019 | 117 | ≤65: 62 (70%) >65: 37 (30%) | 28.1 | NR | CDK4/6+AI n=117 (100%) | AI n=25 (21.4%) Tamoxifen n=5 (4.3%) Fulvestrant n=11 (9.4%) Fulvestrant + CDK4/6i n=3 (2.6%) ET + everolimus n=15 (12.8% Chemotherapy n=54 (46.2%) Unspecified n=4 (3.4%) |
| Nichetti, 2020 | Conference abstract | Retrospective multicenter study | Italy | NR | 25 | NR | NR | NR | CDK 4/6i n=25 (100%) | Everolimus+exemestane n=25 (100%) |
| Crocetti, 2020 | Conference abstract | Single centre retrospective analysis | Italy | Jan 2011 - Dec 2019 | NR | 61 (35-78) | NR | NR | CDK4/6i + ET n=NR | ET +/- Everolimus: n=NR (8.8%) Chemotherapy n=NR (5.8%) |
| Xi, 2019 | Full-text article | Retrospective | US | Feb 2015 - Aug 2017 | 14 | 59.4 (50.7–67.7) | 19.5 (1.7-34.9) | NR | NR | Letrozole n=3 (21.4%) Fulvestrant n=1 (7.1%) Fulvestrant + palbociclib n=1 (7.1%) Exemestane + everolimus n=2 (14.3%) Chemotherapy n=7 (50.0%) |
| Mougalian, 2019 | Full-text article | Retrospective | US | Feb 2015 - Sep 2018 | 121 | Mean: 65.5 (9.9) | Mean (SD): 24.3 (8.7) | 12.2 (NR) | Palbociclib+ET n=102 (84.3%) Palbociclib+letrozole n=67 (55.4%) Palbociclib plus fulvestrant n=22 (18.2%) Palbociclib plus other (e.g., exemestane, anastrozole, etc.) n=13 (10.7%) Ribociclib n=12 (9.9%) Abemaciclib: 6 (5.0%) Palbociclib or abemaciclib without hormonal therapy indicated: 1 (0.8%) | Chemotherapy n=121 (100%) |
| Luhn, 2019 | Conference abstract | Retrospective | NR | Jan 2015 - Oct 2017 | 88 | Mean: 65 (NR) | NR | 9.7 (NR) | Palbociclib n= 88 (100%) | Fulvestrant n=88 (100%) |
| Giridhar, 2019 | Conference abstract | Retrospective single-Institution Study | US | Jun 2014 - Oct 2017 | 37 | 1L CDK4/6: 61 (52 - 68) 2L CDK 4/6: 65 (53 - 73) | NR | NR | NR | Exemestane+everolimus n=10 (27.1%) Chemotherapy n=8 (21.6%) ET n=11 (29.7%) Unspecified n=8 (21.6%) |
| Bashour, 2017 | Full-text article | Case series | US | NR | 4 | 42, 49, 38, 50 | NR | 11.7 (NR) | CDK4/6i+AI n=4 (100%) | Exemestane + Everolimus n=3 (75.0%) Chemotherapy n=1 (25.0%) |

Abbreviations: 1L = first line; 2L = second line; AI = aromatase inhibitors (anastrozole, letrozole, and exemestane); CDK4/6i = cyclin-dependent kinase 4 and 6 inhibitors (palbociclib, ribociclib, and abemaciclib); ET = endocrine therapy including AIs (anastrozole, letrozole, and exemestane), or selective estrogen receptor modulators (tamoxifen and toremifene), or selective estrogen receptor degraders (fulvestrant), or hormones; NR = not reported; PARPi, = poly (ADP-ribose) polymerase inhibitors; UK = United Kingdom; US = United States.

^a^Mean or median of age of the entire patient cohort or patients who failed CDK4/6i as the first line.

**Table S4. Outcomes reported across included real-world evidence studies**

| **Study** | **Type of publication** | **PFS** | | | **TTP** | **OS** | | | | **Response** | | | | | | **PRO** | **Adverse event** | | | | **Treatment duration** | **Treatment modification** | **Treatment discontinuation** |
| --- | --- | --- | --- | --- | --- | --- | --- | --- | --- | --- | --- | --- | --- | --- | --- | --- | --- | --- | --- | --- | --- | --- | --- |
|  |  | **Median PFS** | **6 mos** | **12 mos** |  | **Median OS** | **Death** | **6 mos** | **12 mos** | **ORR** | **CRR** | **PRR** | **Duration** | **SDR** | **CBR** |  | **Overall** | **Grade 3-4** | **TEAE** | **Discontinuation due to AE** |  |  |  |
| Moscetti, 2022 | Conference abstract | ✔ | NR | NR | NR | NR | NR | NR | NR | NR | NR | NR | NR | NR | NR | NR | NR | NR | NR | NR | NR | NR | NR |
| Menichetti, 2022 | Conference abstract | ✔ | NR | NR | NR | NR | NR | NR | NR | NR | NR | NR | NR | NR | NR | NR | NR | NR | NR | NR | NR | NR | NR |
| Marschner, 2022 | Conference abstract | ✔ | NR | NR | NR | NR | NR | NR | NR | NR | NR | NR | NR | NR | NR | NR | NR | NR | NR | NR | NR | NR | NR |
| Kalinsky, 2022 | Conference abstract | ✔ | NR | NR | NR | NR | NR | NR | NR | NR | NR | NR | NR | NR | NR | NR | NR | NR | NR | NR | NR | NR | NR |
| Gousis, 2022 | Conference abstract | ✔ | NR | NR | NR | NR | NR | NR | NR | NR | NR | NR | NR | NR | NR | NR | NR | NR | NR | NR | NR | NR | NR |
| Nichetti, 2020 | Conference abstract | ✔ | NR | NR | NR | NR | NR | NR | NR | NR | NR | NR | NR | NR | NR | NR | NR | NR | NR | NR | NR | NR | NR |
| Crocetti, 2020 | Conference abstract | ✔ | NR | NR | NR | NR | NR | NR | NR | NR | NR | NR | NR | NR | NR | NR | NR | NR | NR | NR | NR | NR | NR |
| Luhn, 2019 | Conference abstract | NR | NR | NR | ✔ | NR | NR | NR | NR | NR | NR | NR | NR | NR | NR | NR | NR | NR | NR | NR | NR | NR | NR |
| Martin, 2022 | Full-text article | ✔ | NR | NR | NR | ✔ | NR | NR | NR | NR | NR | NR | NR | NR | NR | NR | NR | NR | NR | NR | NR | NR | NR |
| Endo, 2022 | Full-text article | NR | NR | NR | NR | NR | NR | NR | NR | NR | NR | NR | ✔ | NR | NR | NR | NR | NR | NR | NR | NR | NR | NR |
| Choong, 2022 | Full-text article | ✔ | NR | NR | NR | NR | NR | NR | NR | NR | NR | NR | NR | NR | NR | NR | NR | NR | NR | ✔ | NR | ✔ | NR |
| Rozenblit, 2021 | Full-text article | NR | NR | NR | ✔ | ✔ | NR | NR | NR | NR | NR | NR | NR | NR | NR | NR | NR | NR | NR | NR | NR | NR | NR |
| Li, 2021 | Full-text article | ✔ | NR | NR | NR | NR | NR | NR | NR | NR | NR | NR | NR | NR | NR | NR | NR | NR | NR | NR | NR | NR | NR |
| Amaro, 2021 | Full-text article | ✔ | NR | NR | NR | NR | NR | NR | NR | NR | NR | NR | NR | NR | NR | NR | NR | NR | NR | NR | NR | NR | NR |
| Xi, 2019 | Full-text article | ✔ | NR | NR | NR | NR | NR | NR | NR | NR | NR | NR | NR | NR | NR | NR | NR | NR | NR | NR | NR | NR | NR |
| Mougalian, 2019 | Full-text article | ✔ | ✔ | NR | NR | NR | ✔ | NR | NR | ✔ | ✔ | ✔ | ✔ | ✔ | ✔ | NR | ✔ | ✔ | NR | ✔ | ✔ | NR | ✔ |
| Bashour, 2017 | Full-text article | NR | NR | NR | ✔ | ✔ | ✔ | NR | NR | NR | NR | NR | NR | NR | NR | NR | NR | NR | NR | NR | NR | NR | NR |
| Giridhar, 2019 | Poster | NR | NR | NR | ✔ | NR | ✔ | NR | NR | NR | NR | NR | NR | NR | NR | NR | NR | NR | NR | NR | NR | NR | NR |

Abbreviations: PFS = Progression-Free Survival; TTP = Time to Progression; TTNT = Time to Next Treatment; OS = Overall Survival; TEAE = Treatment-Emergent Adverse Events; PRO = Patient-Reported Outcomes; ORR = Objective Response Rate; CRR = Complete Response Rate; PRR = Partial Response Rate; SDR = Stable Disease Rate; CBR = Clinical Benefit Rate; Mos = months.

**Table S5. List of studies that lack sufficient information to classify patients based on ESMO/NCCN guidelines**

| **Study Name** | **Total number of patients** | **Number of patients per treatment used in 2L** | **Duration (months)** |
| --- | --- | --- | --- |
| ***rwPFS*** | | | |
| Amaro et al., (2021) | 59 | 25 AI, 5 tamoxifen, 15 everolimus + exemestane, 11 fulvestrant, 3 fulvestrant + CDK4/6i rechallenge | 4.0^a^ |
| Marschner et al., (2022) | 113 | 79 chemotherapy, 22 ET, 10 CDK4/6i rechallenge, 2 PARPi | 4.3 |
| Menichetti et al., (2022) | NR^b^ | Treatment not defined | 5.3 |
| Kalinsky et al., (2022) | 94 | Non-CDK4/6i treatment, not further defined | 8.4 |
| Choong et al., (2022) | 22 | 17 everolimus + exemestane; 1 everolimus + fulvestrant, 4 alpelisib + fulvestrant | 8.5 |
| Li et al., (2021) | 13 | ET ± chidamide, everolimus, or palbociclib | 13.4 |
| Xi et al., (2019) | 7 | 3 letrozole, 2 everolimus + exemestane, 1 fulvestrant, 1 palbociclib + fulvestrant | 17.0 |
| ***DOR*** | | | |
| Endo et al., (2022) | 14 | 5 chemotherapy, 4 ET, 3 everolimus + exemestane, 2 CDK4/6i | 4.3^c^ |

^a^ Although this study reports this information as TTP, it has been extracted as PFS since the definition includes death, not only progression.

^b^ Study did not report sample size.

^c^ Data extracted using DigitizeIt software from the original research paper. Three patients included in this result had a continued response to treatment at the study endpoint.

Abbreviations: AI = aromatase inhibitor; CDK4/6i = cyclin-dependent kinase 4 and 6 inhibitor; DOR = duration of response; ESMO = European Society for Medical Oncology; ET = endocrine therapy; NCCN = National Comprehensive Cancer Network; NR = not reported; PARPi = poly-ADP ribose polymerase inhibitor; PFS = real-world progression-free survival.
